# Pseudo-acetylation of multiple sites on human Tau proteins alters Tau phosphorylation and microtubule binding, and ameliorates amyloid beta toxicity

**DOI:** 10.1038/s41598-017-10225-0

**Published:** 2017-08-30

**Authors:** Marianna Karina Gorsky, Sylvie Burnouf, Oyinkan Sofola-Adesakin, Jacqueline Dols, Hrvoje Augustin, Carina Marianne Weigelt, Sebastian Grönke, Linda Partridge

**Affiliations:** 1 Max Planck Institute for Biology of Ageing, Joseph-Stelzmann-Strasse 9b, 50931 Cologne, Germany; CECAD Cologne Excellence Cluster on Cellular Stress Responses in Aging Associated Diseases, 50931 Cologne, Germany; 2 Institute of Healthy Ageing, and GEE, UCL, Darwin Building, Gower Street, London, WC1E 6BT UK

## Abstract

Tau is a microtubule-associated protein that is highly soluble and natively unfolded. Its dysfunction is involved in the pathogenesis of several neurodegenerative disorders including Alzheimer’s disease (AD), where it aggregates within neurons. Deciphering the physiological and pathogenic roles of human Tau (hTau) is crucial to further understand the mechanisms leading to its dysfunction *in vivo*. We have used a knock-out/knock-in strategy in *Drosophila* to generate a strain with hTau inserted into the endogenous fly *tau* locus and expressed under the control of the endogenous fly *tau* promoter, thus avoiding potential toxicity due to genetic over-expression. hTau knock-in (KI) proteins were expressed at normal, endogenous levels, bound to fly microtubules and were post-translationally modified, hence displaying physiological properties. We used this new model to investigate the effects of acetylation on hTau toxicity *in vivo*. The simultaneous pseudo-acetylation of hTau at lysines 163, 280, 281 and 369 drastically decreased hTau phosphorylation and significantly reduced its binding to microtubules *in vivo*. These molecular alterations were associated with ameliorated amyloid beta toxicity. Our results indicate acetylation of hTau on multiple sites regulates its biology and ameliorates amyloid beta toxicity *in vivo*.

## Introduction

Dysfunction and aggregation of highly post-translationally modified, microtubule-binding Tau proteins is a hallmark of many neurodegenerative diseases including Alzheimer’s disease (AD), the most prominent Tauopathy. However, the molecular mechanisms leading to Tau dysfunction and accumulation are still not entirely understood and, to date, no treatment has been successfully developed to prevent, halt or cure AD. Importantly, the vast majority of Tauopathy cases, including AD, occur sporadically without any mutation in the MAPT gene (for review see ref. 1). Yet, most of the animal models that have been developed to model Tau pathology, both in rodents and in invertebrates such as the fruitfly *Drosophila*, are based on ectopic expression of human Tau (hTau) transgenes (for review see ref. 2). These models can be highly informative, but they often strongly over-express normal hTau proteins, or make use of pathogenic *tau* mutations observed in familial Tauopathies, such as P301L or ΔK280^3^. These models have greatly contributed to the understanding of neurotoxic effects and molecular alterations induced by multiple pathogenic forms of hTau proteins, but they cannot, by nature, recapture some important aspects of sporadic AD, such as the impact of post-translational modifications (PTMs) on hTau function or the binding of hTau to microtubules under physiological conditions. Hence, it is of particular importance to generate in parallel, models of hTau pathology with normal, endogenous expression levels, to decipher the physiological roles of hTau without the drawback of artificial over-expression. One way to generate such models is to use a targeted knock-out/knock-in strategy, to ensure that the human gene is expressed under the control of the endogenous promoter of the orthologous gene in the model organism.

Recently, a new hTau PTM, reversible lysine (K) acetylation has been highlighted. Lysine acetylation is observed within hTau aggregates in AD brains, with so far 4 acetylated lysines being identified, namely K174, K274, K280 and K281^4–8^. Interestingly, the first *in vivo* studies suggested a strong impact of acetylation at these epitopes on hTau turnover, leading to toxicity and neurodegeneration^5, 9^, impaired synaptic plasticity and memory loss^8^. Further, *in vitro* evidence suggests that hTau acetylation on multiple lysines could regulate hTau-induced toxicity^4, 10, 11^, modulating its aggregation propensity and regulating its microtubule bundling function^4, 10^. More than 27 hTau acetylation sites have been identified *in vitro* so far^12^, suggesting that the impact of acetylation on hTau regulation and toxicity could be much broader. Therefore, we generated a new *Drosophila* model of hTau KI, where hTau was expressed under the control of the endogenous *Drosophila tau* promoter, thereby allowing the study of hTau biology and toxicity under *in vivo* physiological conditions. The model was generated by inserting into a *tau* knock-out (KO) fly line^13^ the full-length hTau open reading frame. Using this approach, we achieved hTau expression levels and pattern that matched those of the endogenous fly Tau proteins. hTau KI proteins bound to fly microtubules and were both phosphorylated and acetylated, hence displaying physiological characteristics. Using this new *Drosophila* hTau KI model, we studied the effects of lysine acetylation on hTau-induced toxicity. We simultaneously mutated 4 lysine residues on hTau that were previously shown to be acetylated *in vitro* using mass spectrometry^4, 11^ and that were shown to regulate microtubule bundling *in vitro*, namely K163, K280, K281 and K369^4^. Lysines were mutated to either glutamine (hTau-4Q), to mimic acetylation, or to arginine (hTau-4R), to mimic de-acetylated lysine residues. We observed that KI flies expressing the 4Q acetyl-mimic mutant form of hTau displayed a strikingly different hTau phosphorylation pattern and microtubule-binding affinity from flies expressing the wt- or the 4R-hTau forms, but hTau solubility remained unaffected. Interestingly, pseudo-acetylation of hTau significantly ameliorated amyloid beta (Aβ) toxicity. Altogether, our results suggest that hTau multi-acetylation modulates hTau function and phosphorylation, and leads to ameliorated Aβ toxicity.

## Results

**Figure 1:**
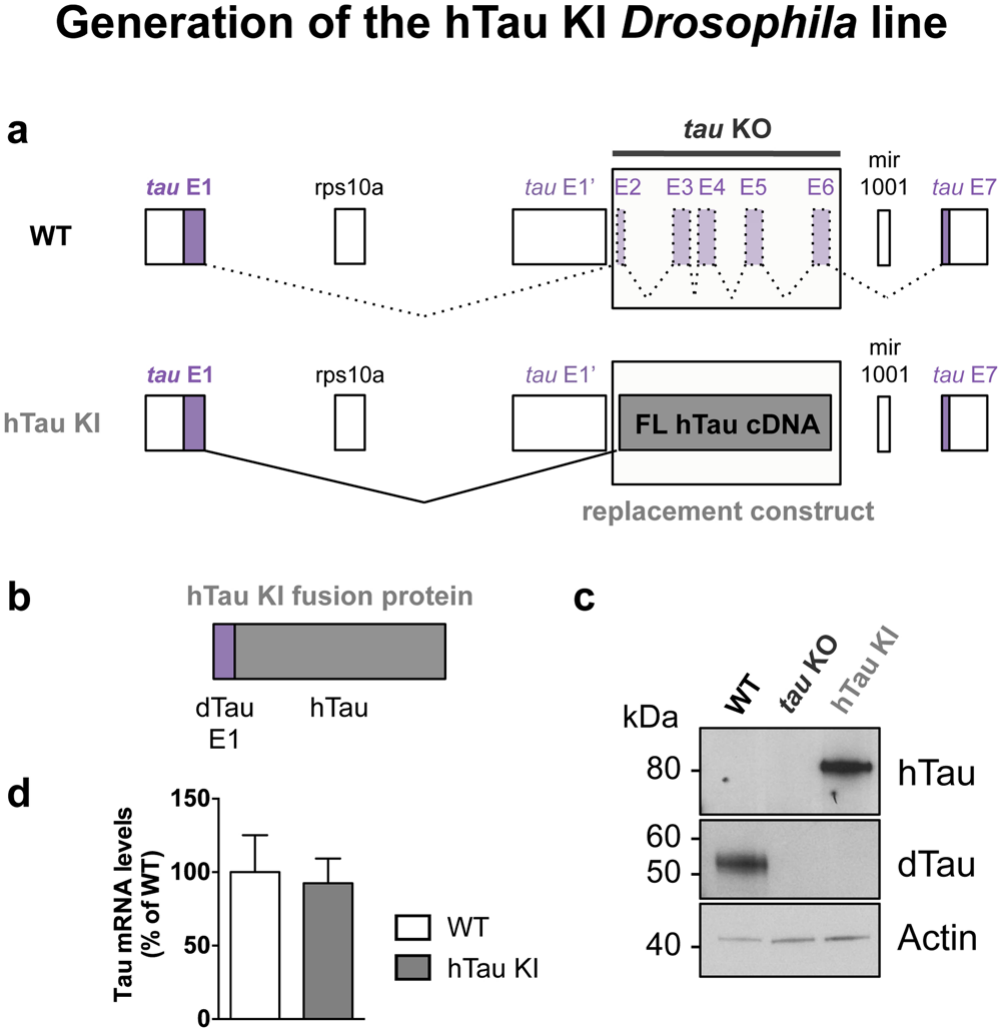
Generation of the hTau KI Drosophila line. (**a**) Schematic representation of the genetic region encompassing the *tau* gene in *Drosophila*. Coding exons of *Drosophila tau* are represented in purple. hTau full-length (2N4R) cDNA sequence (grey) was inserted in place of *tau* exons 2–6 to generate the hTau KI fly line. A more detailed representation is shown in Supplementary Figure 1. (**b**) The expected hTau KI protein product is a fusion of dTau exon 1 (purple) and hTau (grey) (**c**). Western blot analysis of head extracts from WT, *tau* KO and hTau KI flies using anti-hTau and anti-dTau antibodies, indicated that hTau was expressed following its knock-in into the endogenous fly *tau* locus. The apparent molecular weight of the hTau KI protein was 80 kDa. No dTau proteins were expressed in homozygous hTau KI flies. Actin is shown as a loading control (western blots were cropped in this figure; full blots are shown in Supplementary Figure 15). (**d**) Tau mRNA levels measured by qRT-PCR in heads of 20-day-old hTau KI flies were comparable to those of WT flies (p > 0.05, Student’s t-test, n = 3–5 biological replicates). Primers were designed to detect fly *tau* exon 1, present in both fly lines.

**Figure 2:**
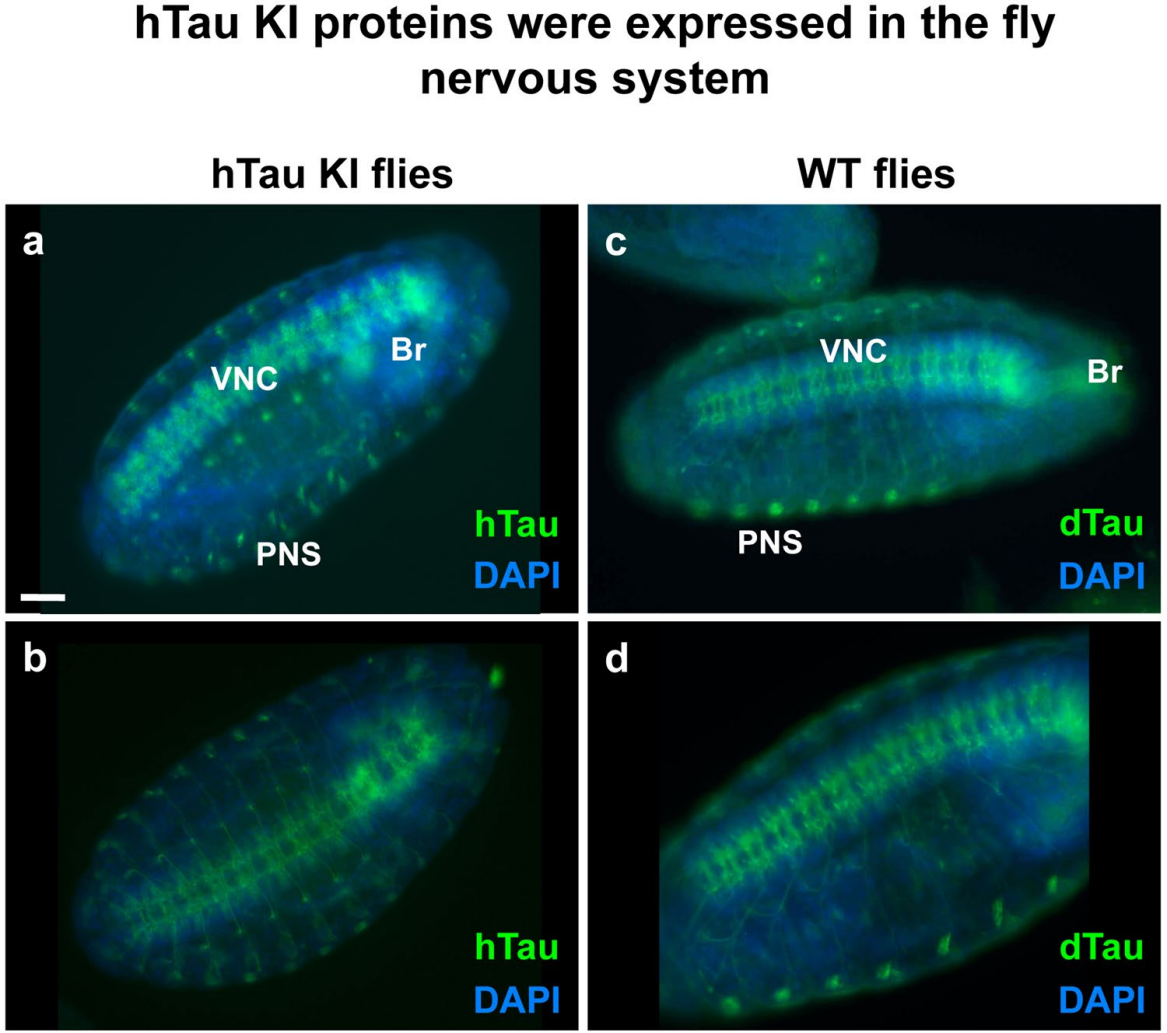
hTau KI proteins were expressed in the fly nervous system. Immunofluorescence on hTau KI fly embryos (**a**,**b**) indicated that hTau was localised to the fly nervous system (Br: brain, VNC: ventral nerve cord, PNS: peripheral nervous system), thereby showing a comparable expression pattern to dTau proteins (**c**,**d**) in WT embryos. Scale bar: 50 µm.

**Figure 3:**
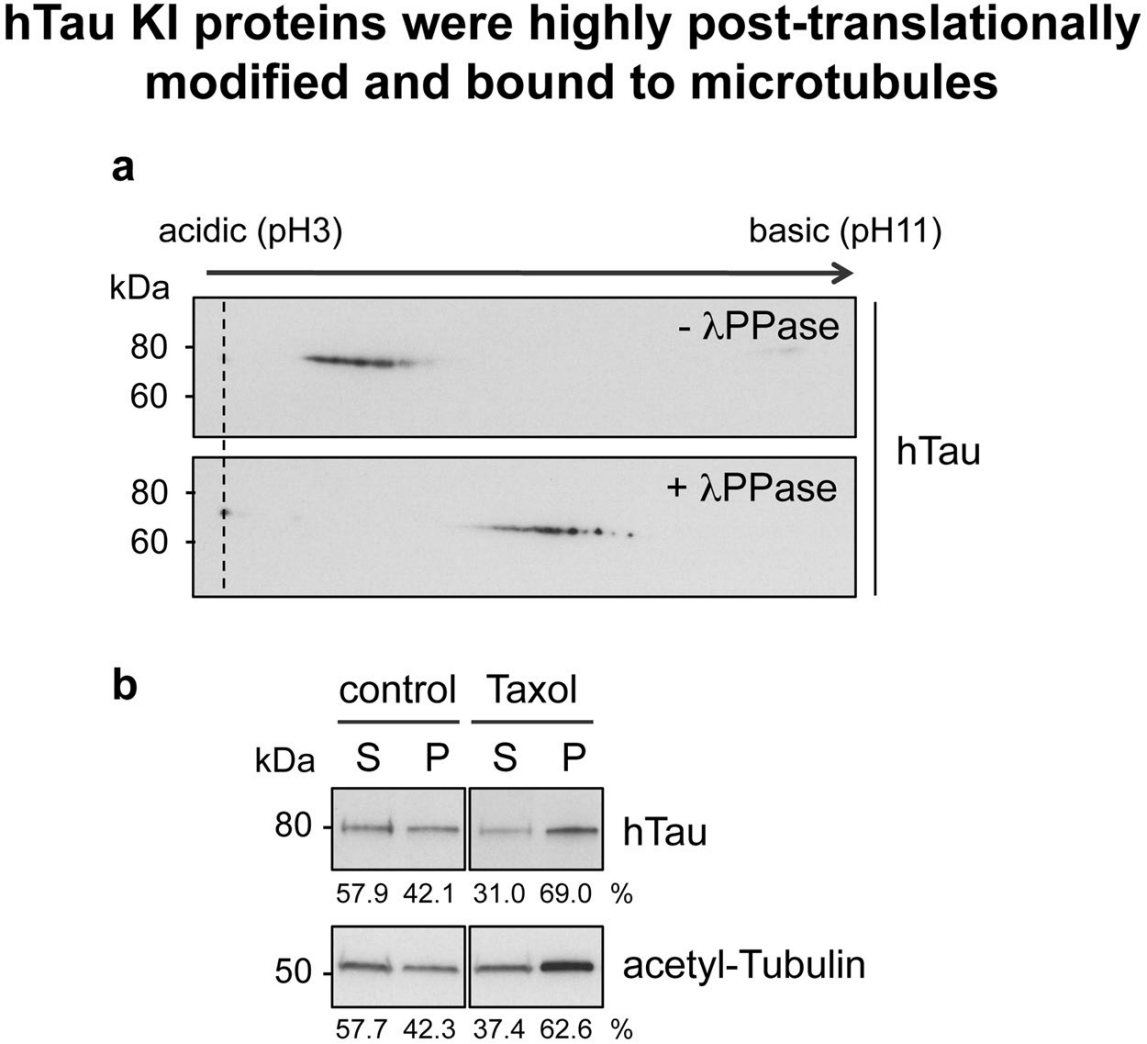
hTau KI proteins were highly post-translationally modified and bound to microtubules. (**a**) 2D gel electrophoresis was performed on total protein extracts retrieved from hTau KI *Drosophila* heads (upper panel). hTau isovariants were detected using an anti-hTau antibody. Lambda phosphatase (λPPase) treatment of the protein extracts resulted in a drastic shift of hTau protein species towards basic pH (lower panel). (**b**) An *in vivo* microtubule-binding assay indicated that hTau proteins bound to microtubules in hTau KI *Drosophila* heads. Incubating protein samples with Taxol induced Tubulin polymerisation, as indicated by the increased amounts of acetyl-Tubulin retrieved in the pelleted fraction. hTau KI proteins precipitated with acetylated Tubulin in Taxol condition. The vehicle, i.e. DMSO, was used for control experiments. S: supernatant, P: pellet. Western blots were cropped in this figure; full blots are shown in Supplementary Figure 16.

To generate a *Drosophila* strain expressing human Tau, we used the previously described *Drosophila tau* KO line^13^, which specifically lacks *tau* exons 2–6, to knock in the cDNA sequence of the hTau full-length 2N4R isoform (Fig. 1a and Supplementary Figure 1). The resulting protein was a fusion between a small N-terminal *Drosophila* Tau (dTau) sequence encoded by exon 1, and hTau (Fig. 1b and Supplementary Figure 2), and had an apparent molecular weight of 80kDa as observed by western blot using an anti-hTau antibody (Fig. 1c). Of note, homozygous hTau KI flies did not express endogenous dTau proteins (Fig. 1c) and were homozygous viable. Since hTau expression should now be regulated by the endogenous dTau promoter, we determined whether hTau KI proteins were expressed in the fly nervous system in a similar way to the endogenous fly Tau proteins. We observed comparable levels of Tau expression in heads of wild type (WT) and hTau KI flies, by qRT-PCR using primers targeting the fly *tau* exon 1 that is expressed in both fly lines (Fig. 1d). In addition, immunofluorescence analysis of hTau KI fly embryos indicated that hTau proteins were expressed in the brain, the ventral nerve cord (VNC) and the central nervous system (CNS) (Fig. 2a and b). This expression pattern was very similar to that of dTau proteins in WT fly embryos, observed using an anti-dTau antibody (Fig. 2c and d). Correspondingly, hTau was expressed in the brain of adult hTau KI flies (Supplementary Figure 3a) in a similar fashion as dTau proteins in WT brains (Supplementary Figure 2b). The specificity of the anti-hTau and anti-dTau antibodies was verified in embryos and brains of control flies (Supplementary Figure 3c and d and Supplementary Figure 4). To further characterise hTau species expressed in the hTau KI model, we performed 2D gel electrophoresis on head extracts from adult hTau KI flies (Fig. 3a, upper panel). Interestingly, western blot analysis using an anti-total-hTau antibody revealed the presence of several hTau isovariants in hTau KI fly heads, characterised by different isoelectrical points. To determine whether these variants were the result of varying patterns of phosphorylation, a post-translational modification that modulates protein charge, we performed a phosphatase treatment on hTau KI protein extracts prior to 2D electrophoresis. This resulted in a pronounced shift of hTau species towards a more basic pH (Fig. 3a, lower panel). These results indicate that, even though hTau proteins were not over-expressed, they were post-translationally modified and highly phosphorylated.

**Figure 4:**
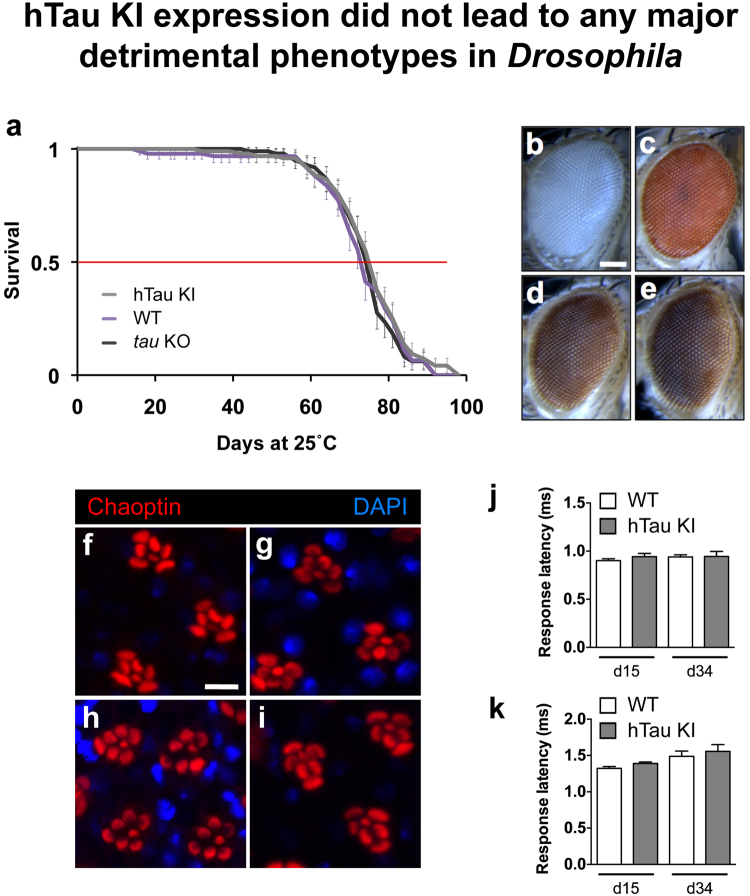
hTau KI expression did not lead to any major detrimental phenotypes in Drosophila. (**a**) The lifespan of hTau KI flies (grey) was comparable to that of both WT (purple) and *tau* KO (black) control flies (p > 0.05, log-rank test). (**b**–**e**) Representative eye pictures of 3-day-old WT (**b**), *tau* KO (**c**) and hTau KI (**d**) flies, and of 22-day-old hTau KI flies (**e**). Scale bar: 100 µm. (**f**–**i**) Evaluation of photoreceptor degeneration in fly retinas was performed using immunofluorescence against Chaoptin (red) in 3-day-old WT (**f**), *tau* KO (**g**) and hTau KI (**h**) flies, and in 22-day-old hTau KI flies (**i**). DAPI was used to stain cell nuclei (blue). Scale bar: 5 µm. (**j** and **k**). Electrophysiology recordings in the TTM (**j**) and DLM (**k**) were performed in 15- and 34-day-old hTau KI flies and showed no significant difference in neurotransmission through the giant fiber neuronal system between hTau KI flies and WT controls (p > 0.05, Student’s t-test).

We investigated whether hTau KI proteins performed their physiological functions in the fly. As hTau is a microtubule-binding protein, we evaluated whether hTau KI proteins could bind to fly microtubules using an *in vivo* microtubule-binding assay^14^. We observed that hTau KI proteins precipitated together with acetylated Tubulin, a marker for polymerised microtubules (Fig. 3b), suggesting that the microtubule-binding function of hTau proteins was preserved in our system. Since hTau expression is usually associated with toxicity (see review ref. 2), we evaluated whether hTau KI expression had detrimental effects on fly survival or induced neuronal degeneration. Interestingly, hTau KI flies displayed a similar lifespan to WT and *tau* KO flies (Fig. 4a, p > 0.05, log-rank test), indicating that hTau expression was not toxic for fly survival. We then analysed, both in fly eyes and brains, whether the expression of hTau KI proteins led to neuronal degeneration. We first evaluated fly eye morphology to assess the potential development of a rough eye phenotype due to hTau expression. However, we did not observe any eye roughening in either young (Fig. 4d) or old (Fig. 4e) hTau KI flies, similar to the eyes of WT and *tau* KO controls (Fig. 4b and c). In addition, using anti-Chaoptin immunofluorescence to specifically stain rhabdomeres from fly retinas, we did not observe any loss of photoreceptor neurons either in eyes of 3-day-old hTau KI flies (Fig. 4h) compared to age-matched WT (Fig. 4f) and *tau* KO (Fig. 4g) or in eyes of old hTau KI flies (Fig. 4i). We next evaluated the levels of the Elav neuronal protein and of two synaptic proteins, Synapsin and Syntaxin, in brain extracts from 20-day-old hTau KI flies and did not observe any alteration of their levels compared to age-matched WT flies (Supplementary Figure 5), suggesting no neurotoxic effect of hTau expression. Finally, to evaluate whether neuronal function was impaired upon hTau expression, we carried out electrophysiological recordings from the giant fiber system to quantitatively assess the functional status of this pathway^15^ (Fig. 4j and k). This well-defined neuronal circuit is involved in mediating rapid escape behaviour and was previously shown to deteriorate upon expression of toxic proteins^16^. We measured response latency i.e. the time for the electric signal to reach either the tergotrochanteral (TTM, Fig. 4j) or the dorsal longitudinal muscle (DLM, Fig. 4k), following stimulation of the giant fiber in the fly brain. We did not detect any alteration of neurotransmission within this circuit in the hTau KI line, in either 15- or 34-day-old flies (Fig. 4j and k). Thus, the new hTau KI fly model produced functional hTau proteins that were expressed in the normal, endogenous pattern, and that were post-translationally modified by phosphorylation. Furthermore, hTau KI proteins bound to fly microtubules, did not lead to overt neurodegeneration and were not toxic to nervous system functions.

**Figure 5:**
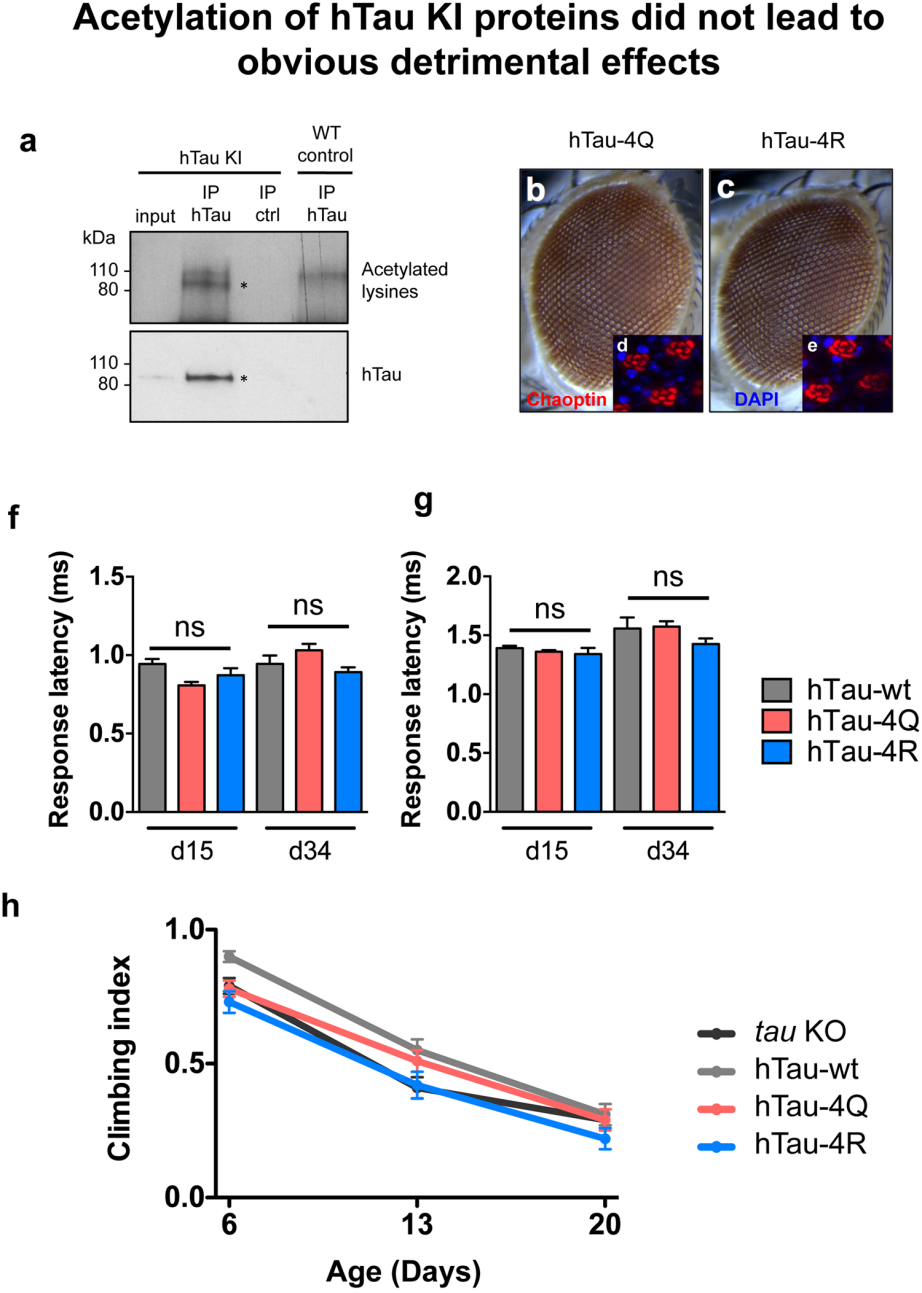
Acetylation of hTau KI proteins did not lead to obvious detrimental effects. (**a**) hTau proteins were acetylated in hTau KI *Drosophila*. Immunoprecipitation of hTau followed by western blot against acetylated lysines (upper panel) and total hTau proteins (lower panel) revealed a specific band for acetylated hTau species (asterisk), indicating that hTau proteins were acetylated in hTau KI fly heads. hTau immunoprecipitation on protein extracts from WT flies was performed as a negative control. Western blots were cropped in this figure; full blots are shown in Supplementary Figure 17. (**b** and **c**) Representative eye pictures of 3-day-old hTau-4Q (**b**) and hTau-4R (**c**) flies. Scale bar: 100 μm. (**d** and **e**) Photoreceptor loss in fly retinas was evaluated by anti-Chaoptin immunofluorescence (red) in 3-day-old hTau-4Q (**d**) and hTau-4R (**e**) KI flies. DAPI was used to stain cell nuclei (blue). Scale bar: 5 μm. (**f** and **g**) Electrophysiology recordings in the TTM (**f**) and DLM (**g**) was performed in 15- and 34-day-old hTau-wt, hTau-4Q and hTau-4R KI flies and showed no significant difference in neurotransmission through the giant fiber neuronal system among the investigated lines (p > 0.05, one-way ANOVA). (**h**) Climbing analysis was performed on hTau-wt, hTau-4Q, hTau-4R and *tau* KO control flies throughout ageing, showing no detrimental effect of hTau expression on fly climbing.

Next, we investigated whether hTau KI proteins were acetylated. We found that hTau immunoprecipitation from *Drosophila* heads, followed by western blotting targeting acetylated lysines, revealed the presence of acetylated hTau species in hTau KI flies (Fig. 5a), suggesting that Tau acetylation is a conserved event in *Drosophila*. We therefore used our hTau KI model to further investigate, *in vivo* and under endogenous conditions, the impact of acetylation on hTau function. We selected 4 previously detected acetylation sites from two independent *in vitro* studies^4, 11^, namely K163, K280, K281 and K369 (Supplementary Figure 6). We generated hTau KI fly lines expressing either pseudo-acetylated (hTau-4Q) or pseudo-de-acetylated (hTau-4R) mimic mutants of these sites in the full-length hTau protein. Of note, the non-mutated “hTau KI” line will be named “hTau-wt” in the following experiments. As expected from such a KI system, hTau mRNA levels (Supplementary Figure 7a and b) were comparable among the three KI lines, allowing unbiased analysis of the functional consequences directly attributable to the generated hTau protein isoforms. We then performed immunofluorescence experiments to confirm proper hTau protein expression in embryos (Supplementary Figure 8, left panels) and adult brains (Supplementary Figure 8, right panels) of hTau-4Q and hTau-4R KI flies, and observed a similar pattern of hTau expression in their tissues to that in hTau-wt flies. To probe the biological consequences of mimicry of altered patterns of acetylation, we first evaluated neuronal degeneration by analysing eyes of hTau-4Q and hTau-4R flies (Fig. 5b and c). We did not observe any major detrimental effect of expression of these hTau species, as hTau-4Q and hTau-4R KI flies developed no rough eye phenotype, similar to hTau-wt flies (Fig. 4d). We then performed an anti-Chaoptin staining on retinas of 3-day-old hTau-4Q (Fig. 5d) and hTau-4R (Fig. 5e) KI flies and observed no loss of photoreceptor neurons, similar to what we observed following hTau-wt expression (Fig. 4h). Next, we evaluated neuronal function of these fly strains by means of electrophysiological recordings from the giant fiber system and observed no significant detrimental or beneficial effect of hTau-4Q or hTau-4R expression compared to hTau-wt on neurotransmission in the TTM (Fig. 5f, p > 0.05, one-way ANOVA) or in the DLM (Fig. 5g, p > 0.05, one-way ANOVA). We analysed neuronal function of the hTau KI lines through evaluation of fly climbing ability but did not observe any detrimental effect of the expression of any of these hTau species on climbing behaviour compared to *tau* KO control flies (Fig. 5h, p > 0.05, two-way ANOVA).

**Figure 6:**
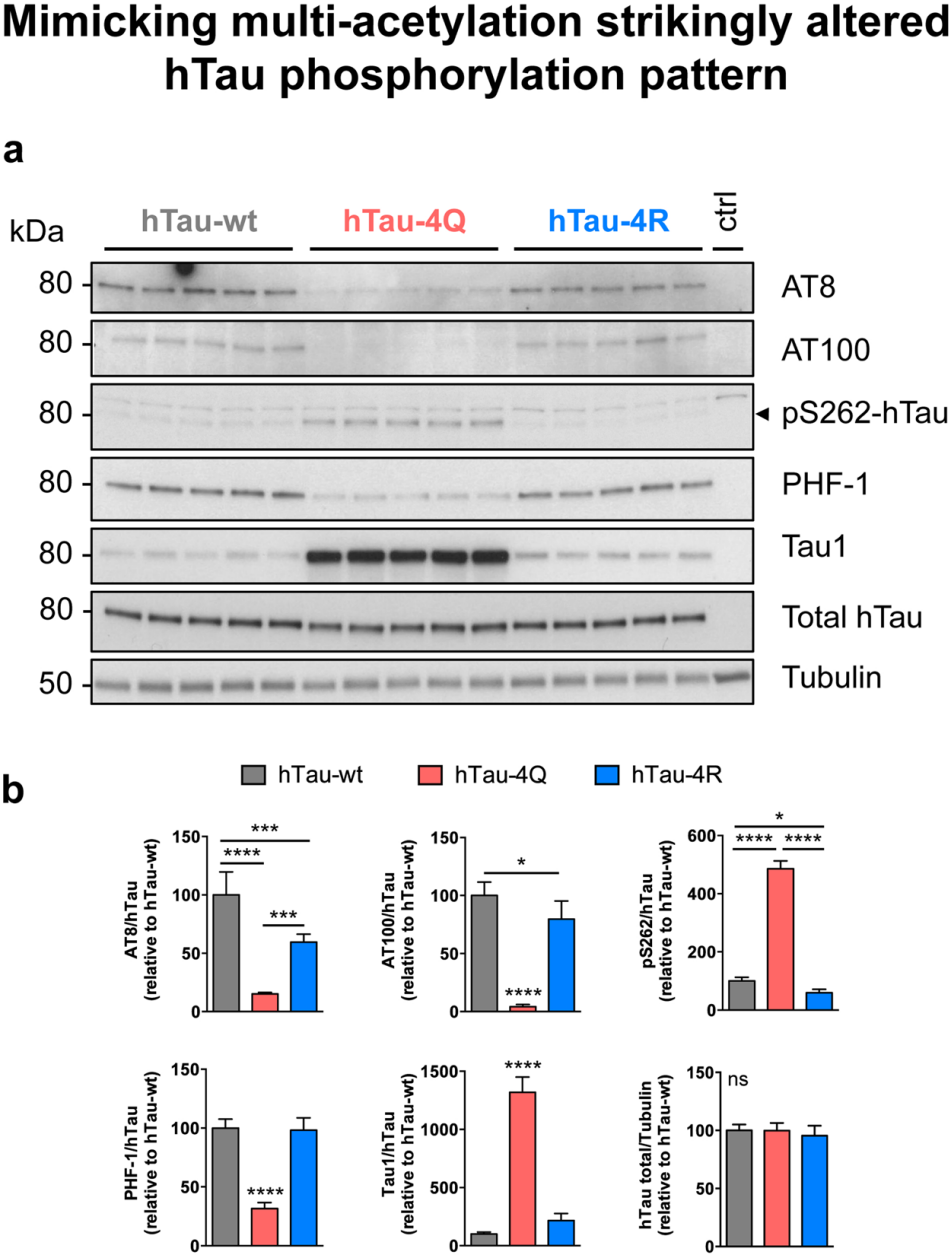
Mimicking multi-acetylation strikingly altered hTau phosphorylation pattern. Western blot analysis (**a**) and quantification (**b**) of hTau phosphorylation (AT8, AT100, anti-pS262-hTau, PHF-1 and Tau1 antibodies) and total hTau levels (polyclonal K9JA antibody) retrieved from head extracts of 2-day-old hTau-wt, hTau-4Q and hTau-4R KI flies. The arrow indicates the specific band for the pS262-hTau western blot. Results were normalised to Tubulin and to total hTau levels and were expressed relative to levels observed in the hTau-wt KI line (*p < 0.05, ***p < 0.001 and ****p < 0.0001, one-way ANOVA followed by Tukey’s post hoc test, n = 5/genotype). Western blots were cropped in this figure. Full blots are shown in Supplementary Figure 18.

We also assessed the effects of hTau pseudo-acetylation status on hTau phosphorylation. Although total hTau levels were not altered by their pseudo-acetylation status (p > 0.05, one-way ANOVA, Fig. 6a and b), hTau phosphorylation was strikingly altered. We analysed a total of 11 hTau phosphorylation sites covered by 5 antibodies (Supplementary Figure 6) i.e. Tau1 (dephosphorylated S195, S198, S199 and S202), AT8 (pS202/pT205), AT100 (pT212/pS214), pS262 and PHF-1 (pS396/pS404). While phosphorylation at S262 was specifically increased in hTau-4Q mutants (****p < 0.0001, vs. hTau-wt and hTau-4R, one-way ANOVA, Fig. 6a and b), all of the 10 other investigated phosphorylation sites were severely dephosphorylated compared to both hTau-wt and hTau-4R expressing flies (***p < 0.001 and ****p < 0.0001, one-way ANOVA, Fig. 6a and b). Notably, this effect was also observed in older flies (Supplementary Figure 9). Next, we analysed hTau phosphorylation in pseudo-acetylated or pseudo-de-acetylated forms of a single hTau epitope, i.e. K280 (Supplementary Figure 10). Besides AT100 phosphorylation, which was significantly decreased in the hTau-K280Q fly line (*p < 0.05 vs. hTau-wt and ***p < 0.001 vs. hTau-4R, one-way ANOVA), we did not observe any significant modulation of hTau phosphorylation following mutation of the single hTau-K280 residue. Since both acetylation and phosphorylation events have been shown to regulate hTau solubility *in vitro* ^4, 10, 17^, we analysed whether hTau-wt, hTau-4Q and hTau-4R species displayed different solubility *in vivo*. However, we did not observe any significant alteration of hTau solubility in our hTau KI lines following fractionation using a combination of RAB and RIPA-1% SDS buffers (p > 0.05. one-way ANOVA, Supplementary Figure 11a and b).

**Figure 7:**
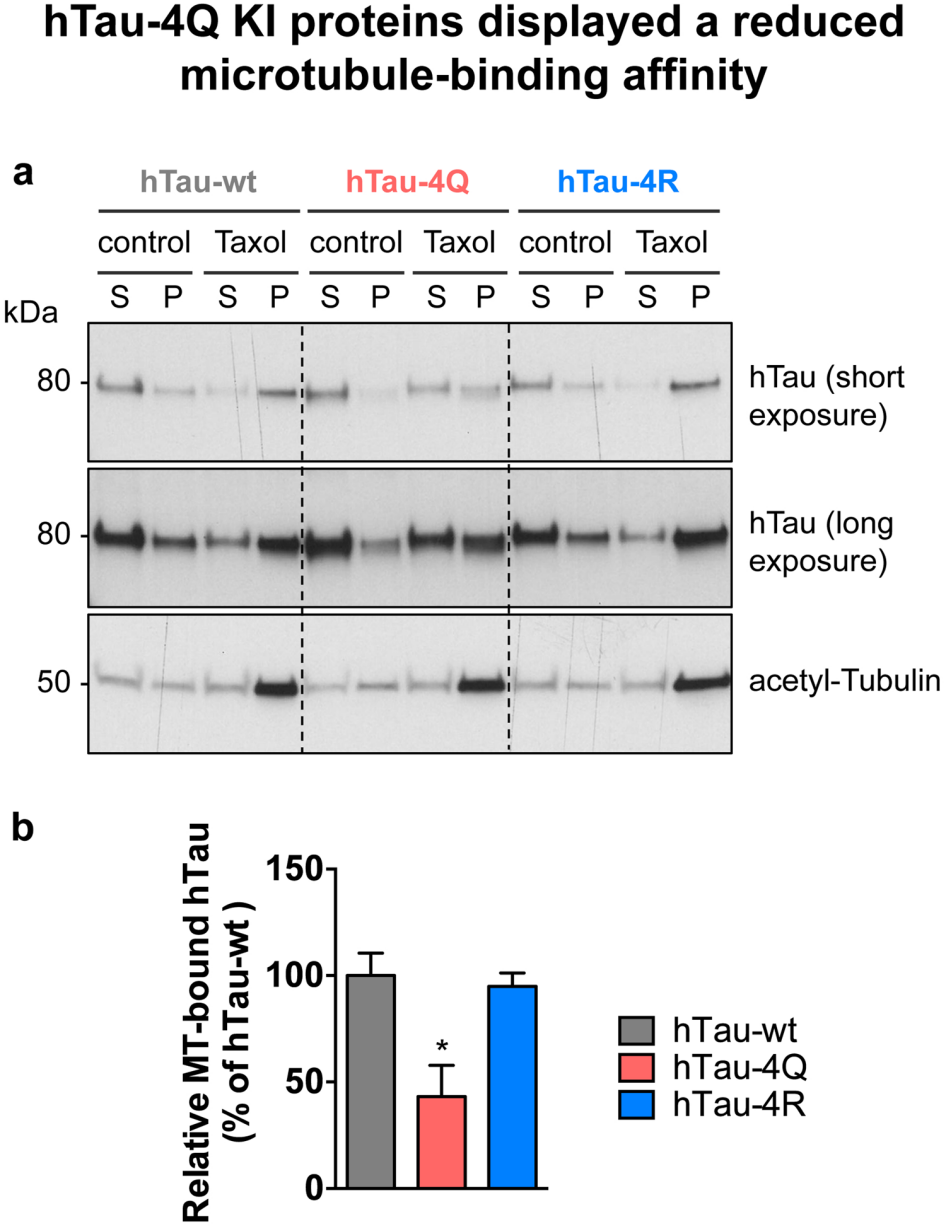
hTau-4Q KI proteins displayed reduced microtubule-binding affinity. (**a**) *In vivo* microtubule-binding assay was performed on protein extracts from 2-day-old hTau-wt, hTau-4Q and hTau-4R KI fly heads. Taxol was used to induce microtubule polymerisation, and DMSO, the vehicle, was used as a control. S: supernatant, P: pellet. Western blots were cropped in this figure; full blots are shown in Supplementary Figure 19. (**b**) Quantification of the proportion of microtubule-bound hTau proteins in hTau-wt, hTau-4Q and hTau-4R KI lines, in Taxol conditions. Results are expressed relative to levels measured in hTau-wt KI flies (*p < 0.05, one-way ANOVA followed by Tukey’s post hoc test, n = 3/genotype).

hTau microtubule-binding function is regulated by its charge, and is therefore influenced by post-translational modifications^18, 19^, thus, we evaluated whether hTau pseudo-acetylation or pseudo-de-acetylation impaired its microtubule-binding properties (Fig. 7). Surprisingly, we observed that hTau-4Q species, despite a globally reduced phosphorylation status (Fig. 6), were less abundant in the fraction enriched for polymerised microtubules than both hTau-wt and hTau-4R isoforms (*p < 0.05, one-way ANOVA, Fig. 7a and b). These data suggest that pseudo-acetylated hTau-4Q species were less efficient at binding to microtubules than hTau-wt or pseudo-de-acetylated hTau. This effect did not translate into decreased levels of total acetyl-Tubulin, as observed in head extracts from hTau-4Q KI flies compared to hTau-wt and hTau-4R KI flies (p > 0.05, one-way ANOVA, Supplementary Figure 12). We also analysed microtubule binding of the hTau-K280Q and hTau-K280R species (Supplementary Figure 13). We observed no significant difference in microtubule binding ability of these hTau species compared to hTau-wt proteins (p > 0.05, one-way ANOVA).

**Figure 8:**
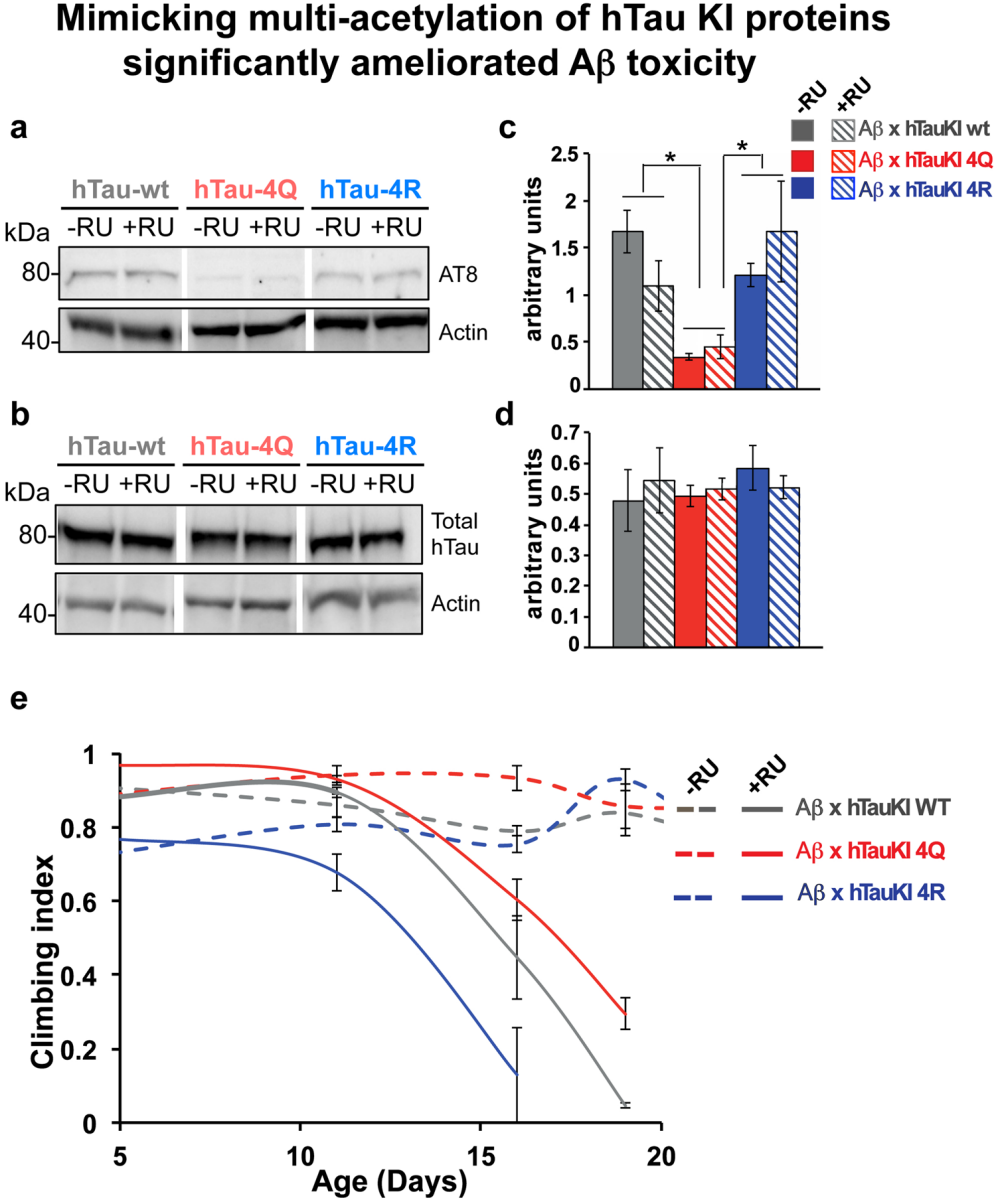
Mimicking multi-acetylation of hTau KI proteins ameliorated Aβ toxicity. (**a**) Western blot analyses (**a** and **b**) and quantification (**c** and **d**) of hTau phosphorylation (AT8) and total hTau levels were carried out respectively on head extracts of 12 day-old flies either co-expressing Aβ (+RU) with the different homozygous hTau KI lines, hTau-wt, hTau-4Q and hTau-4R, or expressing homozygous hTau KI lines alone (−RU), grown at 28 °C. Results were normalised to Actin. (n = 3/genotype, *p < 0.05, one-way ANOVA followed by Tukey’s post hoc test). Western blots were cropped in this figure. Full blots are shown in Supplementary Figure 20. (**e**) Climbing assay shows that the flies co-expressing Aβ (+RU) and homozygous hTau-4Q line have improved climbing (n = 3, *p < 0.05, beta regression analyses) and flies co-expressing Aβ (+RU) and homozygous hTau-4R line climb worse (n = 3, ****p < 0.0001, beta regression analyses) in comparison to control flies co-expressing Aβ (+RU) and homozygous hTau-wt. Homozygous hTau KI lines (−RU) alone did not differ in their climbing ability. Genotype of flies expressing A**β**42 arc in the different hTauKI homozygous backgrounds, A**β**42 arc/+; hTauKI (wt, 4R or 4Q)/elavGS,hTauKI (wt, 4R or 4Q).

Finally, we used our hTau KI lines to gain insight into the interaction of amyloid beta (Aβ) and Tau acetylation, and investigated whether acetylated hTau KI lines altered Aβ toxicity *in vivo*. We made use of the previously generated inducible model, that expresses arctic mutant Aβ42 solely in adult neurons and causes toxicity when expressed in adult fly neurons by the elav gene switch (elavGS) driver^16, 20^. We crossed the flies expressing arctic Aβ42 with the different hTau KI flies, and assessed the phosphorylation pattern, and climbing behaviour of the progeny. First, we measured the levels of Aβ42 across the three groups of flies co-expressing arctic Aβ42 in combination with any of the hTauKI lines (A**β**42 arc/+; hTauKI/elavGS,hTauKI), and observed similar levels of Aβ42 protein (p > 0.05, one-way ANOVA, Supplemental Figure 14). Next, we measured the levels of total Tau, and of phosphorylated Tau at the AT8 site, which is associated with AD pathogenesis^21^. We found both that the striking pattern of hTau phosphorylation at the AT8 site was maintained, similar to flies expressing hTau KI lines alone (Fig. 8a and c), and that total hTau levels were not changed (Fig. 8b and d). Flies expressing either hTau-4Q mutants alone or co-expressing arctic Aβ42 and hTau-4Q mutants were severely dephosphorylated at the AT8 site, compared to flies expressing either hTau-wt and hTau-4R, with or without arctic Aβ42 (*p < 0.05, one-way ANOVA, Fig. 8a and c). We went on further to determine whether pseudo-acetylation of Tau was associated with any phenotypic consequences in the arctic Aβ42 expressing flies. Interestingly, we found that flies co-expressing hTau-4Q with arctic Aβ42 showed an improved climbing behaviour in comparison to control flies, co-expressing arctic Aβ42 and hTau-wt (*p < 0.05, beta regression). On the other hand, hTau-4R significantly worsened arctic A**β**42 toxicity in comparison to control flies co-expressing arctic Aβ42 and hTau-wt (****p < 0.0001, beta regression), (Fig. 8e).

Altogether, our results indicate that the simultaneous pseudo-acetylation of K163, K280, K281 and K369 drastically alters hTau phosphorylation, modulates hTau microtubule-binding properties *in vivo*, and unexpectedly ameliorates A**β** toxicity significantly.

## Discussion

Model organisms are powerful systems in which to study and understand the function and dysfunction of hTau proteins *in vivo*. Using a knock-out/knock-in strategy, we have generated a new *Drosophila* model to investigate hTau function under physiological conditions *in vivo*. We used this model to evaluate the effects of hTau multi-acetylation, and found an effect on hTau microtubule-binding and phosphorylation. Furthermore, hTau multi-acetylation was associated with improved Aβ toxicity *in vivo*.

We generated hTau KI lines by introducing the full-length 2N4R hTau cDNA sequence into the gene locus of its fly homologue, i.e. *Drosophila tau*. We took advantage of the previously generated *Drosophila tau* KO line, which does not express any microtubule-binding dTau isoforms^13^. The resulting hTau KI was a fusion protein holding exon 1 of dTau on its N-terminal end. In contrast to commonly used tags including GFP and mCherry, dTau exon 1 is relatively small (82 amino acids for 8.84 kDa) and is an endogenous fly component, hence mitigating potential artificial toxic effects and abnormal localisation and functions of hTau proteins that may result from a fusion with an exogenous tag. However, whether dTau exon 1 influences hTau biology will deserve further experiments. By using a KI system, we ensured that hTau followed the same expression pattern as endogenous dTau proteins. In addition, hTau KI proteins were expressed from embryonic stages and during adulthood, similarly to dTau, further suggesting that our knock-in strategy was successful.

We observed that hTau KI proteins bound to microtubules when expressed under physiological conditions. This result is in contrast with a previous study reporting a very weak microtubule-binding of over-expressed 2N4R hTau proteins in *Drosophila*, both in the presence or absence of dTau^14^, and suggests that the knock-in strategy was more effective than over-expression systems for investigating this aspect of hTau function. In addition, we observed that hTau KI proteins were highly phosphorylated, further indicating that knocked-in hTau proteins were regulated in accordance with their intrinsic phospho-protein characteristics. Interestingly, hTau KI expression did not result in detectable toxic effects either on fly lifespan or on neuronal degeneration in either fly eyes or brains, or on neuronal function. These observations contrast with the toxic effects obtained following hTau over-expression in *Drosophila* (for review see ref. 2), and suggest that expressing physiological, endogenous levels of human Tau protein is essentially harmless *in vivo*. This new fly tool will therefore enable future studies investigating potential toxic effects triggered following expression of mutant forms of hTau-KI proteins. Homozygous hTau KI flies did not express dTau proteins, hence separating the effects related to hTau expression from those of the endogenous dTau.

Following on from our previous study indicating that removal of dTau proteins does not lead to detrimental effects in *Drosophila* ^13^, we did not observe any eye roughening or loss of photoreceptor neurons in the *tau* KO fly line. Hence the evaluation of these phenotypes cannot be used to assess a potential compensation of dTau loss of function by hTau KI expression. However, because we observed the binding of hTau proteins to fly microtubules, our results suggest that hTau is indeed functional in this KI system.

Interestingly, 2D gel electrophoresis performed on hTau KI fly heads following incubation with lambda phosphatase suggested that other post-translational modifications than phosphorylation were present. Immunoprecipitation experiments indicated that these proteins were also acetylated. We therefore used our KI system to investigate the impact of multi-site acetylation on hTau function, with a focus on K163, K280, K281 and K369, previously shown to be highly acetylated in two independent *in vitro* studies^4, 11^. We generated acetylation- and de-acetylation-mimic mutant forms of hTau to investigate the functional consequences of simultaneously modulating these 4 lysine residues *in vivo*. First, we observed a striking crosstalk between hTau acetylation and phosphorylation. While phosphorylation on S262 was increased in the hTau-4Q acetylation-mimic mutant, all other investigated hTau phosphorylation sites were severely dephosphorylated. Since hTau is an unstructured protein^22^, it is likely that acetylation events change hTau conformation, as do phosphorylation and other post-translational modifications^23, 24^. Such conformational change, in turn, might have exposed some hTau phospho-epitopes, on one hand, and regulated the binding of kinases and phosphatases to hTau, on the other, thus modifying hTau phosphorylation pattern. In support of our data, a recent paper by Trzeciakiewicz *et al*. presented similar findings. They showed *in vitro* that cells co-expressing WT Tau and the acetyltransferase CREB-binding protein led to marked Tau dephosphorylation at several sites – AT8, AT270, AT180, and pS262. Furthermore, they showed that expressing either hTau-4Q or a double lysine deletion (ΔK280/ΔK281) also reduced phosphorylation at AT8 and pS262 sites, which was attributed to increased access of the phosphatase PP2A to unbound hTau^25^. However, there were slight differences from our results, since we observed increase in pS262 levels in the hTau-4Q expressing flies, highlighting possible differences between *in vitro* and *in vivo* environment. Similar to other published results in mice over-expressing Aβ in a wild-type humanized Tau background^26^, we did not observe Arctic Aβ42-induced changes in the phosphorylation pattern of the hTau KI lines.

Previous studies have reported a detrimental effect of hTau-4Q species on microtubule bundling in cell cultures^4^. In accordance with this observation, we found that hTau-4Q KI proteins bound to fly microtubules with a lower efficiency than both hTau-wt and hTau-4R isoforms *in vivo*. As mentioned above, it is likely that pseudo acetylation on multiple sites causes conformational changes, by possibly neutralising the positive charges of the lysines^27^, which are likely to induce modifications in hTau localisation, protein-protein interactions and phosphorylation *in vivo*, ultimately potentially regulating its bioavailability for microtubules. It is also possible that the specific increase in S262 phosphorylation, which adds a negative charge to hTau and is known to impair binding to microtubules^22^, accounts for the decreased affinity of hTau-4Q proteins for microtubules. The single K280Q acetylation-mimic mutation on hTau was also shown to significantly alter microtubule bundling in cell cultures^4, 25^, thus we aimed to investigate whether it was sufficient to reduce hTau binding to fly microtubules *in vivo*. Interestingly, we did not observe any significant influence of this single-site mutation on hTau microtubule binding properties and phosphorylation - except AT100, which was significantly reduced in hTau-K280Q KI mutants. Very little is known so far about the specific effects of hTau acetylation on microtubule binding and on the crosstalk with hTau phosphorylation, and further studies, both *in vitro* and *in vivo*, will be required to investigate these important aspects.

Interestingly, the differences we observed in hTau phosphorylation did not influence hTau protein solubility, because all three hTau protein isoforms were highly soluble. This is in line with previous reports indicating that hTau phosphorylation does not necessarily lead to its aggregation^9, 28^. Further investigation will be required to evaluate whether soluble, oligomeric hTau species are produced in hTau KI flies and whether they contribute to the different phenotypes we observed.

We did not observe any obvious positive or negative modulation of neuronal function in hTau-4Q and hTau-4R KI flies as compared to hTau-wt KI flies. However, expression of hTau-4Q and hTau-4R improved or worsened Aβ toxicity respectively in comparison to hTau-wt KI flies. Several hypotheses can be formulated to explain this effect. First, we observed a drastic overall dephosphorylation of pseudo-acetylated hTau-4Q species *in vivo*. Phosphorylation events within the proline-rich region of hTau have been shown to regulate its binding to SH3 domain-containing interaction partners^29, 30^, thereby potentially regulating their functions within cells. Moreover, hTau phosphorylation status has been reported to regulate hTau subcellular localisation and, in particular, its binding to the plasma membrane^31, 32^. These effects, directly linked to hTau phosphorylation, could induce specific cellular adaptations resulting in ameliorated Aβ toxicity. While we did not detect major changes in terms of hTau protein localisation within the embryonic and adult tissues of hTau-4Q flies compared to hTau-wt and hTau-4R flies, further studies will be required to determine whether the subcellular localisation of hTau-4Q proteins is modified. Second, in line with previous cell culture observations^4^, our results indicated a weaker binding of hTau-4Q proteins to fly microtubules. While this aspect would require further investigation, one could speculate that the pool of unbound hTau-4Q proteins triggered specific signalling pathways that ameliorated Aβ toxicity.

Noteworthy, the hTau-wt KI line showed similar phenotypes to hTau-4R KI flies in terms of microtubule binding and hTau phosphorylation, both being far apart from phenotypes displayed by hTau-4Q flies. Such results suggest that hTau-wt KI proteins behaved as if they would be de-acetylated in a similar way to hTau-4R species. Further experiments will be important to determine to what extent hTau-wt proteins are acetylated *in vivo*.

In summary, we have generated an hTau KI model where hTau proteins were expressed under the control of the endogenous fly *tau* promoter in the fly nervous system, and displayed physiological characteristics such as phosphorylation and microtubule-binding properties. In addition, using this KI system, we observed reduced microtubule-binding affinity and severely altered phosphorylation of acetylated hTau species *in vivo*. Importantly, multi-site pseudo-acetylation of knocked-in hTau proteins unexpectedly led to ameliorated Aβ toxicity. Altogether, our results suggest that acetylation is an important event for the regulation of hTau function in endogenous conditions, and that occurrence of these PTMs on hTau proteins can result in complex effects *in vivo*. Therefore, better understanding of the complex interactions of PTMs on hTau is vital to determine the best approach to target PTMs as potential therapeutic interventions for AD pathogenesis.

## Materials and Methods

### Generation of the hTau knock-in fly lines

We used genomic engineering^33^ and a previously generated *tau* knock-out (KO) *Drosophila* line^13^ to generate the hTau KI strains. The genomic engineering strategy is outlined in Supplementary Figure 1. hTau KI constructs consisted of hTau 2N4R cDNA and sequences from the endogenous fly *tau* locus. Acetylation-mimic and de-acetylation-mimic forms of hTau were designed by replacing lysines at positions 163/280/281/369 with glutamine (hTau-4Q) or arginine (hTau-4R), respectively. Constructs were synthesized by Eurofins Genomics (Germany) in a pGEattB vector^33^. hTau-K280Q and hTau-K280R KI fly lines were designed by mutating hTau at lysine 280 and were generated by site-directed mutagenesis using the QuikChange II Site-Directed Mutagenesis Kit following the manufacturer’s instructions (Agilent Technologies). hTau KI fly lines were obtained using the φC31 and attP/attB targeted integration system and constructs were inserted into the attP landing-site located in the endogenous *tau* locus of *Drosophila tau* founder lines (Supplementary Figure 1). Arctic Aβ42 and elav gene switch (elavGS) driver fly lines have been previously described^20^. Positive fly lines were verified by PCR, sequencing and western blotting. The resulting hTau KI proteins were a fusion of dTau exon 1 (82 amino acids) and hTau.

### Fly stocks and maintenance

All fly stocks were kept at 25 °C in standard conditions as previously described^9^. All fly lines (except hTau-K280Q and hTau-K280R KI lines) were backcrossed into a white Dahomey wild-type (WT), outbred genetic background for at least seven generations prior to experiments. Experiments were carried out with female flies and at 29 °C except otherwise stated.

### Lifespan analysis

150 to 200 once-mated female flies per genotype were allocated to food vials at a density of 10 flies per vial and kept at a temperature of 25 °C. The number of dead flies was recorded every 2–3 days as flies were transferred to fresh food. Lifespan results are expressed as the proportion of survivors ± 95% confidence interval.

### Climbing assay

Analysis of fly climbing was performed blindly as described before^20^. At least 3 biological replicates of 15–20 females flies per genotype were used for the analysis. Flies used for climbing experiments were kept at 28 °C or 29 °C.

### Immunofluorescence in *Drosophila* embryos

This procedure was based on^34^ with some modifications. Briefly, embryos were washed in saline solution (0.7% NaCl and 0.03% Triton x-100) and dechorionated with bleach for 3 min. Following fixation in fixative solution (1 vol of heptane and 1 vol of formaldehyde 4%) for 30 min at room temperature, embryos were incubated for one hour at 4 °C in blocking solution (5% BSA in PBT with 0.2% of Triton X-100) and then subsequently incubated overnight at 4 °C with the anti-dTau antibody (1/1000 in PBT with 5% BSA) or with the anti-hTau K9JA antibody (Dako, 1/5000 in PBT with 5% BSA). Alexa Fluor 488 secondary antibody was used at a dilution of 1/200 in PBT with 5% BSA for two hours at 4 °C. Embryos were mounted in Vectashield with DAPI mounting medium and visualised using a LEICA DMI4000B/DFC 340FX inverted microscope and a 10X objective. Flies used for these experiments were kept at 25 °C.

### Immunofluorescence on fly brains and thoracico-abdominal ganglia

Following dissection in 1X PBS and subsequent fixation in PBS with 4% paraformaldehyde for 15 min, adult fly brains and thoracico-abdominal ganglia were washed three times in PBST (PBS with 0.3% Triton X-100), blocked for one hour in PBST with 5% BSA, and then incubated overnight with anti-human Tau (K9JA) or anti-*Drosophila* Tau antibodies at a dilution of 1/2000 in PBST with 5% BSA. Anti-rabbit Alexa Fluor 488 secondary antibody was used at a dilution of 1/500 in PBST with 5% BSA for one hour. Tissues were mounted in Vectashield with DAPI and images were taken using a Zeiss (UK) LSM 700 confocal laser scanning microscope using a 10x objective.

### Eye phenotyping and evaluation of photoreceptor loss

Eye pictures of 3-day-old and 22-day-old flies were taken using a Leica M165 FC stereomicroscope equipped with a motorized stage and the multifocus tool in the Leica application suite software. Retinas of 3- and 22-day-old flies were dissected in 1X PBS and fixed in 4% paraformaldehyde in PBS for 2 hours at 4 °C. Retinas were washed 6 × 30 min in 1 mL PBT (PBS with 0,5% Triton X-100) at room temperature (RT) and subsequently blocked in 1 mL blocking buffer (PBT with 5% fetal bovine serum and 0,01% Sodium Azide) for 60 min at RT. Anti-Chaoptin antibody (24B10, Developmental Studies Hybridoma Bank) was used at 1/200 in 300 µL blocking buffer and incubated with the retinas overnight at 4 °C. Following 6 washes of 30 min each in PBT, retinas were incubated with goat anti-mouse Alexa Fluor 594 secondary antibody (1/500 in 300 µL blocking buffer) overnight at 4 °C. Retinas were then washed in PBT at RT, incubated in 50% glycerol in PBS for 30 min and subsequently mounted on a microscope slide in Vectashield HardSet Antifade Mounting Medium with DAPI (Vectorlabs). Imaging was done using a Leica SP5-X confocal microscope. Flies used for these experiments were kept at 25 °C.

### Electrophysiology measurements

Recordings from the Giant Fiber circuit were made as described in^13^ and initially described by Tanouye and Wyman^35^. Briefly, activation of the giant fiber was achieved in the fly brain using a tungsten stimulating electrode and recordings were made from the tergotrochanteral muscle (TTM) and the contralateral dorsal longitudinal muscle (DLM) using glass microelectrodes. For response latency recordings, at least 3–5 single stimuli were given with a 5s rest period between each stimulus. Recordings were performed blindly from at least 4 flies per genotype.

### Sample preparation and Western Blotting

20 female heads per biological replicate were homogenized by sonication in 200 μL of RIPA-1% SDS buffer supplemented with Complete mini without EDTA protease inhibitor (Roche). Protein concentration was measured using the BCA protein assay kit (Pierce) according to the manufacturer’s instructions. 5–10 µg of total proteins were supplemented with 2x LDS containing reducing agent (Invitrogen) or female heads were homogenized directly in Laemmli sample buffer containing β-mercaptoethanol, and heated at 98 °C for 10 minutes prior to loading on Any kD Criterion gels (Biorad). Proteins were transferred to nitrocellulose membranes (GE Healthcare) that were subsequently blocked in TNT buffer (Tris–HCl 15 mM pH 8, NaCl 140 mM, 0.05% Tween) or TBST with 5% non-fat dry milk for 1 hr at room temperature and incubated overnight at 4 °C with the following antibodies: anti-dTau (1/10000) ref. 13, anti-dephosphorylated S195/S198/S199/S202-hTau (Tau1, 1/1000, Millipore), pS202/T205-hTau (AT8, 1/2000, Thermo Scientific), anti-pT212/S214-hTau (AT100, 1/2000, Thermo Scientific), anti-pS262-hTau (1/5000, Invitrogen), anti-pS396/S404 (PHF1, 1/2000, Peter Davies), anti-total hTau K9JA (1/100,000, Dako), anti-Elav (1/1000, Developmental Studies Hybridoma Bank (DSHB)), anti-Synapsin (1/500, DSHB), anti-Syntaxin (1/500, DSHB), anti-acetylated-lysines (9441, 1/1000, Cell Signaling), anti-acetyl-Tubulin (D20G3, 1/2000, Cell Signaling), anti-α-Tubulin (11H10, 1/2000, Cell Signaling) and anti-β-Actin (1/200,000, Abcam). HRP-conjugated anti-mouse or anti-rabbit antibodies (1/10,000, Invitrogen) were used for 1 h at room temperature and detection was performed using an ECL chemiluminescence kit (GE Healthcare) and Hyperfilms (GE Healthcare).

### Immunoprecipitation

20 fly heads were extracted by sonication in 100µL of RIPA buffer supplemented with complete mini without EDTA and acetylation inhibitors (10 µM Trichostatin A, Santa Cruz, and 20mM Nicotinamide, Sigma-Aldrich). 100 µg of total proteins were incubated overnight at 4 °C with or without the anti-hTau antibody (1/1000) in a final volume of 100 µL. Next day, samples were incubated with 10 µL of PureProteome Protein G magnetic beads (Millipore) for 10 minutes at room temperature. After several washes, beads were incubated with 15 µL of 2x LDS containing reducing agent (Invitrogen) and heated at 90 °C for 10 minutes. Eluted samples were transferred to clean tubes prior to loading on Any Kd Criterion gels (Biorad).

### *In vivo* microtubule-binding assay

Microtubule-binding experiments were based on previous reports^14^ with some modifications. Briefly, 40 female fly heads were extracted in 150 µL of Buffer-C+ (50 mM HEPES, 1 mM MgCl_2_, 1 mM EGTA, 50 mM NaF, 10 mM Na_3_VO_4_ and complete mini without EDTA) containing either 20 µM of Taxol (Paclitaxel, Sigma-Aldrich) or the same volume of DMSO, the vehicle. After centrifugation at 1000 g for 10 minutes, supernatants were layered on top of 300 µL of buffer-C+ containing 50% sucrose. Following centrifugation at 100,000 g for one hour, 150 µL of the upper fraction (S) containing soluble tubulin was transferred to a fresh tube. Pellets (P) containing polymerised microtubules were resuspended in 150 µL of 2x LDS containing reducing agent (Invitrogen) by pipetting and incubation in a sonication bath for 5 minutes. Protein concentration of the soluble fraction was measured using the BCA system (Pierce). Equal volumes of soluble and insoluble fractions were loaded on Any kD Criterion Gels for analysis. Temperature was kept at 25 °C throughout the experiment. Binding of hTau species to fly microtubules was evaluated by normalising the percentage of pelleted hTau to the percentage of pelleted acetyl-Tubulin, in Taxol conditions.

### Phosphatase treatment

Heads of hTau KI flies were homogenized by sonication in ice-cold RIPA (Pierce) buffer supplemented with Complete mini without EDTA protease inhibitor (Roche). Eighty micrograms of total proteins were treated with Lambda Phosphatase (New England Biolabs) for 3 hours at 37 °C. Samples were stored at −80 °C until use for 2D gel electrophoresis.

### 2D gel electrophoresis

2D gel electrophoresis experiments were performed as previously described^13^ using eighty micrograms of hTau KI fly head protein. Isoelectrofocalisation was performed following the manufacturer’s instructions using Immobiline Drystrips (pH 3–11NL, 11 cm). SDS-PAGE was performed using 10% Bis-Tris IPG + 1 Criterion gels (Biorad) and proteins were transferred to 0.45 µm nitrocellulose membranes prior to antibody staining.

### Soluble-insoluble fractionation

This experiment was performed as in Gorsky *et al*.^9^ using heads of hTau-wt, -4Q and -4R KI flies (20 heads per biological replicate). Tissues were homogenized by sonication in 150 μL ice-cold RAB buffer (Pierce) and Complete mini without EDTA protease inhibitor (Roche) and subsequently centrifuged at 100,000 g for 1 h at 4 °C. The supernatant (“soluble fraction”) was collected and the pellet was homogenized in 150 μL of RIPA-1% SDS by pipetting. Homogenized pellets were centrifuged at 100,000 g for 1 h at RT and the supernatant was collected (“insoluble fraction”). Protein concentration was evaluated in the soluble fraction using the BCA protein assay kit (Pierce). 5 μg of the soluble fraction and twice the amount of the equivalent volume of the insoluble fraction were used for western blotting.

### RNA extraction and qRT-PCR

A Trizol-Chloroform-based protocol (Invitrogen) was used to extract total RNA from heads of female flies (25 heads per replicate). Following treatment with DNAse I (Ambion), 300 ng of RNA were subjected to cDNA synthesis using the SuperScript Vilo Mastermix (Invitrogen). Quantitative real-time PCR was performed using TaqMan primers targeting either exon 1 of *Drosophila tau* or a hTau sequence common to the three hTau-wt/4Q/4R KI lines (Applied Biosystems) in a 7900HT real-time PCR system (Applied Biosystems). The relative expression of *tau* was determined by the ΔΔ*C*_T_ method and was normalised to *actin5c*. Three to five independent biological replicates per group were analysed. Results are plotted as mean ± SEM.

### Elisa

Quantification of Aβ1–42 was carried out as previously described^16^. To extract total Aβ, five *Drosophila* heads were homogenised in 50 µl GnHCl extraction buffer (5 M Guanidine HCl, 50 mM Hepes pH 7.3, protease inhibitor cocktail (Sigma, P8340) and 5 mM EDTA), centrifuged at 21,000 g for 5 min at 4 °C, and cleared supernatant retained as the total fly Aβ sample. Aβ was measured using the hAmyloid β42 ELISA kit (IBL INTERNATIONAL). N of 4 individual samples were diluted in sample/standard dilution buffer and ELISA performed according to the manufacturers’ instructions. Protein extracts were quantified using the Bradford protein assay (Bio-Rad protein assay reagent; Bio-Rad laboratories (UK) Ltd) and the amount of Aβ in each sample expressed as a ratio of the total protein content (pmol/g total protein).

### Statistical analysis

For lifespan experiments, statistical differences were assessed using the log-rank test. Other results are expressed as mean ± SEM and differences between mean values were determined using either Student’s *t* test, one-way or two-way ANOVA followed by Tukey’s post-hoc test, and beta regression analyses using either Graphpad Prism software, JMP 12 or R. *p* values < 0.05 were considered significant.

## Electronic supplementary material

Supplementary information
