# Supplementary material for: Pseudo-acetylation of multiple sites on human Tau proteins alters Tau phosphorylation and microtubule binding, and ameliorates amyloid beta toxicity
Source: Sci Rep. 2017 Aug 30;7:9984. doi: 10.1038/s41598-017-10225-0 (PMC5577152; doi:10.1038/s41598-017-10225-0)
Supplement: Supplementary file 1 — Supplementary information [file 41598_2017_10225_MOESM1_ESM.pdf]

Supplementary Figures

Pseudo-acetylation of multiple sites on human Tau proteins alters Tau phosphorylation and microtubule binding, and ameliorates amyloid beta toxicity.

Marianna Karina Gorsky[1*], Sylvie Burnouf[1*], Oyinkan Sofola-Adesakin[2*], Jacqueline Dols[1], Hrvoje Augustin[1,2],, Carina Marianna Weigelt[1], Sebastian Grönke[1] and Linda Partridge[1,2**]

[1]Max Planck Institute for Biology of Ageing, Joseph-Stelzmann-Strasse 9b, 50931 Cologne, Germany; CECAD Cologne Excellence Cluster on Cellular Stress Responses in Aging Associated Diseases, 50931 Cologne, Germany.
[2]Institute of Healthy Ageing, Department of Genetics, Evolution and Environment, University College London, Darwin Building, Gower Street, London, WC1E 6BT, UK.

*Authors contributed equally to this manuscript

**Correspondence should be addressed to L. P. (Linda.Partridge@age.mpg.de; Telephone: 0049(0)221-37970602; Fax: 0049(0)221-3797088602).

Supplementary Figure-1-Partridge. Genomic engineering of the fly *tau* locus

a

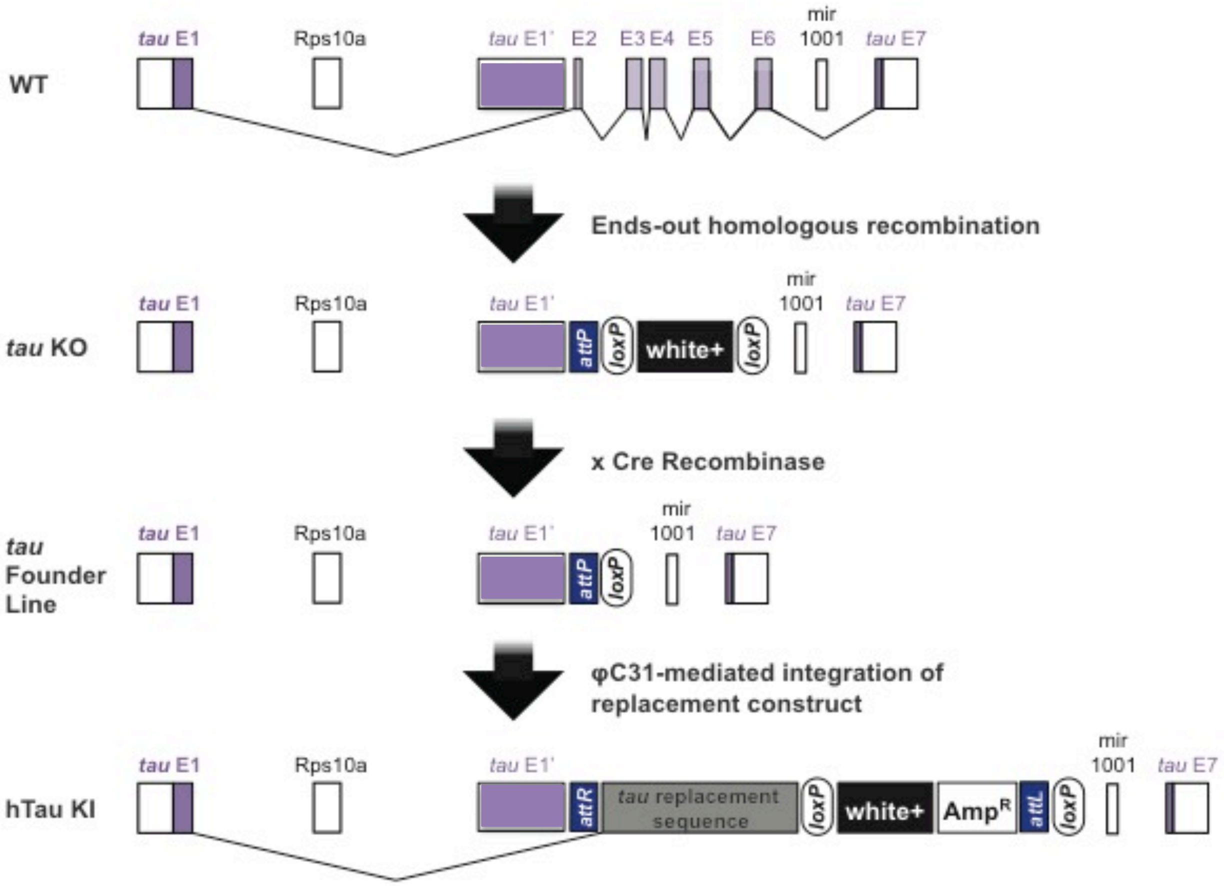

b

GAATTCAGTTTATTTTTAAAATTTAACTATCTCAATATGATTCATGATATATCCATCCATGCCTCCCTCATGTATCTTTTGGTTTATTTCAGAGAT
GGCTGAGCCCCGCCAGGAGTTCGAAGTGATGGAAGATCACGCTGGGACGTACGGGTTGGGGGACAGGAAAGATCAGGGGGGCTAC
ACCATGCACCAAGACCAAGAGGGTGACACGGACGCTGGCCTGAAAGAATCTCCCCTGCAGACCCCCACTGAGGACGGATCTGAGGA
ACCGGGCTCTGAAACCTCTGATGCTAAGAGCACTCCAACAGCGGAAGATGTGACAGCACCCTTAGTGGATGAGGGAGCTCCCGGCA
AGCAGGCTGCCGCGCAGCCCCACACGGAGATCCCAGAAGGAACCACAGCTGAAGAAGCAGGCATTGGAGACACCCCCAGCCTGGA
AGACGAAGCTGCTGGTCACGTGACCCAAGCTCGCATGGTCAGTAAAAGCAAAGACGGGACTGGAAGCGATGACAAAAAAGCCAAG
GGGGCTGATGGTAAAACGAAGATCGCCACACCGCGGGGAGCAGCCCCTCCAGGCCAG**AAG**GGCCAGGCCAACGCCACCAGGATTC
CAGCAAAAACCCCGCCCGCTCCAAAGACACCACCCAGCTCTGGTGAACCTCCAAAATCAGGGGATCGCAGCGGCTACAGCAGCCCC
GGCTCCCCAGGCACTCCCGGCAGCCGCTCCCGCACCCCGTCCCTTCCAACCCCACCCACCCGGGAGCCCAAGAAGGTGGCAGTGG
TCCGTACTCCACCCAAGTCGCCGTCTTCCGCCAAGAGCCGCCTGCAGACAGCCCCCGTGCCCATGCCAGACCTGAAGAATGTCAAGT
CCAAGATCGGCTCCACTGAGAACCTGAAGCACCAGCCGGGAGGCGGGAAGGTGCAGATAATTAAT**AAGAAG**CTGGATCTTAGCAAC
GTCCAGTCCAAGTGTGGCTCAAAGGATAATATCAAACACGTCCCGGGAGGCGGCAGTGTGCAAATAGTCTACAAACCAGTTGACCTG
AGCAAGGTGACCTCCAAGTGTGGCTCATTAGGCAACATCCATCATAAACCAGGAGGTGGCCAGGTGGAAGTAAAATCTGAGAAGCTT
GACTTCAAGGACAGAGTCCAGTCGAAGATTGGGTCCCTGGACAATATCACCCACGTCCCTGGCGGAGGAAAT**AAA**AAGATTGAAACC
CACAAGCTGACCTTCCGCGAGAACGCCAAAGCCAAGACAGACCACGGGGCGGAGATCGTGTACAAGTCGCCAGTGGTGTCTGGGG
ACACGTCTCCACGGCATCTCAGCAATGTCTCCTCCACCGGCAGCATCGACATGGTAGACTCGCCCCAGCTCGCCACGCTAGCTGACG
AGGTGTCTGCCTCCCTGGCCAAGCAGGGTTTGTAGATGCCGAAATGCATGTCGAGCTGTATCTGAAACTGAATCTGAAACTGACTCTTTC
TTTGCAAGCTAAGATCAACAAAAGTGAAATATGAGCAGCTAATTTGAAACGTACTATCTAAGCAAAATCCACACAATGCAAAACGAGAGTTA
TGCGATAATTTCCACACACCCAGATAATTCACAAGCCAGTAAATGCGATCAGATCAATTCGATAGAGGTGCAAGCACGCAATGAGATGCAG
ATACTCGTACATCTACACAACCACCCCACATAGAAGCGGTATTTGGTATACAATCGAAAGTATATTTGACTAGTTGAGCAATGCACTCTTTAT
CATCAGATAGCAGACATGAATTATATATCTCAATGTATTACCAGAACTTCATATATACATACAAATCTATATATATATATATATACATATATAAATAAA
AGTATCTAAATATTTAAGCAAATTATACAAGAAACGTACGAATAAAGTGTATGCTTAGTTGAAAATGTTAGTAAACTACGTGTACATAAGCTCA
AAGTAGTTACGTATGATATCTAGAAATTAGCATTAACCCTGATTTATTACTAGACTCTTTATTTGATTAAGAAAGCAACCAAAAGTAATCGATAC
GAGTATATTACAAACATCTATTATCGCTATCCCATTTTGTGTTTACGTCGAGAAATTCATTTTTATGTAATCTGTAATGGTAATCAATTACGTTG
CATTTTCGATTAACACTCATTTATTTTAATATGCAACTAACAGAGCACCACACCCCAAAAGAGGCCCACACAACACCCAAAACACCCAACCA
CCACCCACAGACACGCCCCACCCGAACCGCTTTCAGATAACTCCTACCAACTCCCAAAGATCAATCCGTTTAATGGGGAATTGTGATTATA
AGAACTAACAATTGGACAACGTATTTTGGCACATCTAGGCATCGAATTGTATTTTATTGTGGGCATTCGTGACTTTTATCAGATAGAGCCAC
CGAAATCTTTAGAGATTTGAAACCATCTATGAAGTGTCTAAAAGTATGCTTTGAAAAATAGAAAGTAGCTGCATACCTTTAGACATTTGTTGA
AGTGATTGCAAACCTCTTGAAGTTCCGTAGCTACCATTGATTATTATTTGTTTTTGATTTTAGATGATTTATTGTATTATCTATTGGTTTCTCAG
TTCAATTACACTTACTTTACATTTAAAAATGAATTTCTTTCAAACTCAAACTGAGATTCATTGGCAACAGCGCTTTCCATCTCGAG

HUMAN TAU 2N4R cDNA / K163, 280, 281 and 369 / DROSOPHILA TAU RECONSTITUTION SEQUENCES

Supplementary Figure-2-Partridge. Amino acid sequence of the hTau KI fusion protein.

MADVLEKSSLLDAVPPLGDPHPPLPHQQLQQEAAAAAAANAAPPA

PPQQQQPPPHQLQQQQPQQQQLQQKPANARANQDQKE**MAEPR**

QEFEVMEDHAGTYGLGDRKDQGGYTMHQDQEGDTDAGLKESPL

QTPTEDGSEEPGSETSDAKSTPTAEDVTAPLVDEGAPGKQAAAQ

PHTEIPEGTTAEEAGIGDTPSLEDEAAGHVTQARMVSKSKDGTGS

DDKKAKGADGKTKIATPRGAAPPGQKGQANATRIPAKTPPAPKTP
163

PSSGEPPKSGDRSGYSSPGSPGTPGSRSRTPSLPTPPTREPKKV

AVVRTPPKSPSSAKSRLQTAPVPMPDLKNVKSKIGSTENLKHQPG

GGKVQIINKKLDLSNVQSKCGSKDNIKHVPGGGSVQIVYKPVDLS
280 281

KVTSKCGSLGNIHHKPGGGQVEVKSEKLDFKDRVQSKIGSLDNIT

HVPGGGNKKIETHKLTFRENAKAKTDHGAEIVYKSPVVSGDTSPR
369

HLSNVSSTGSIDMVDSPQLATLADEVSASLAKQGL

DROSOPHILA TAU E1 (82 amino acids)
HUMAN TAU 2N4R (441 amino acids)
LYSINES 163, 280, 281 and 369

Predicted molecular weight: 54.67 kDa
Apparent molecular weight on SDS-PAGE: 80 kDa

Supplementary Figure-3-Partridge. hTau KI proteins were expressed in brains of adult flies.

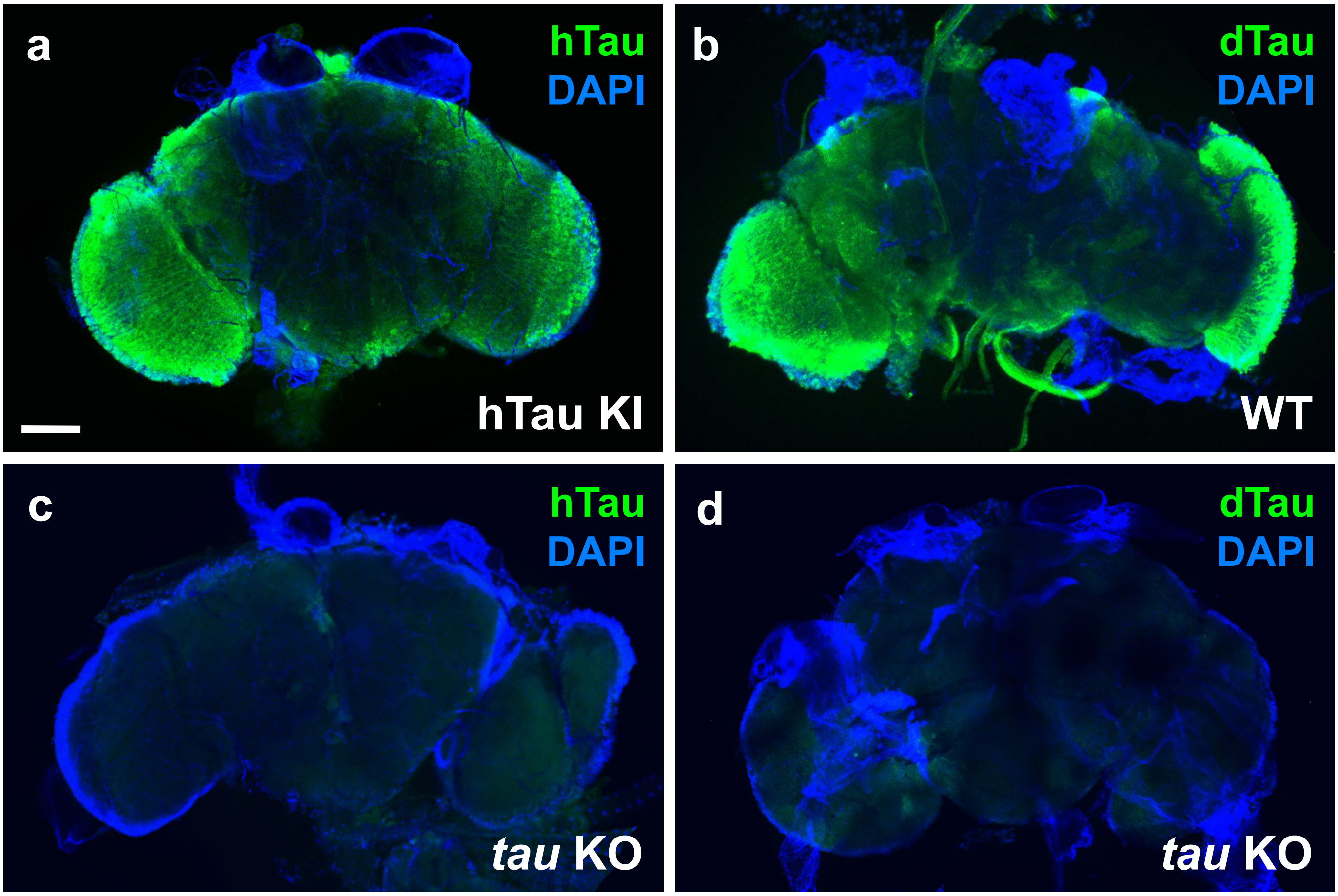

Supplementary Figure-4-Partridge. Control experiments for hTau immunofluorescence on fly embryos.

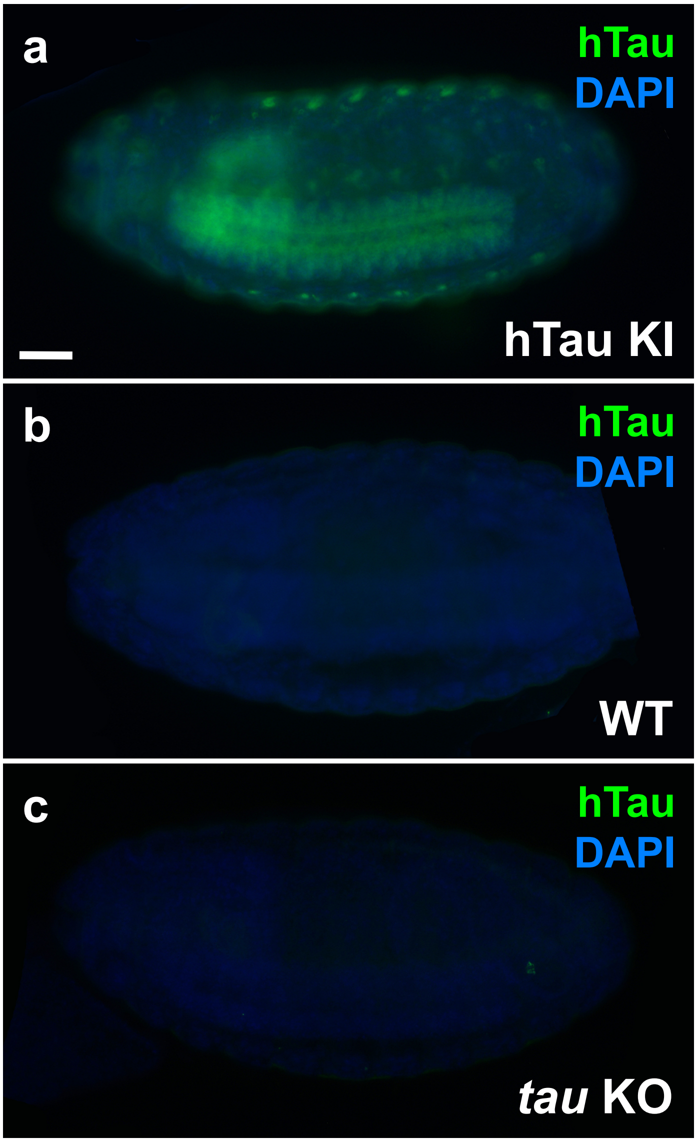

Supplementary Figure-5-Partridge. Neuronal and synaptic protein levels were not altered in hTau KI flies.

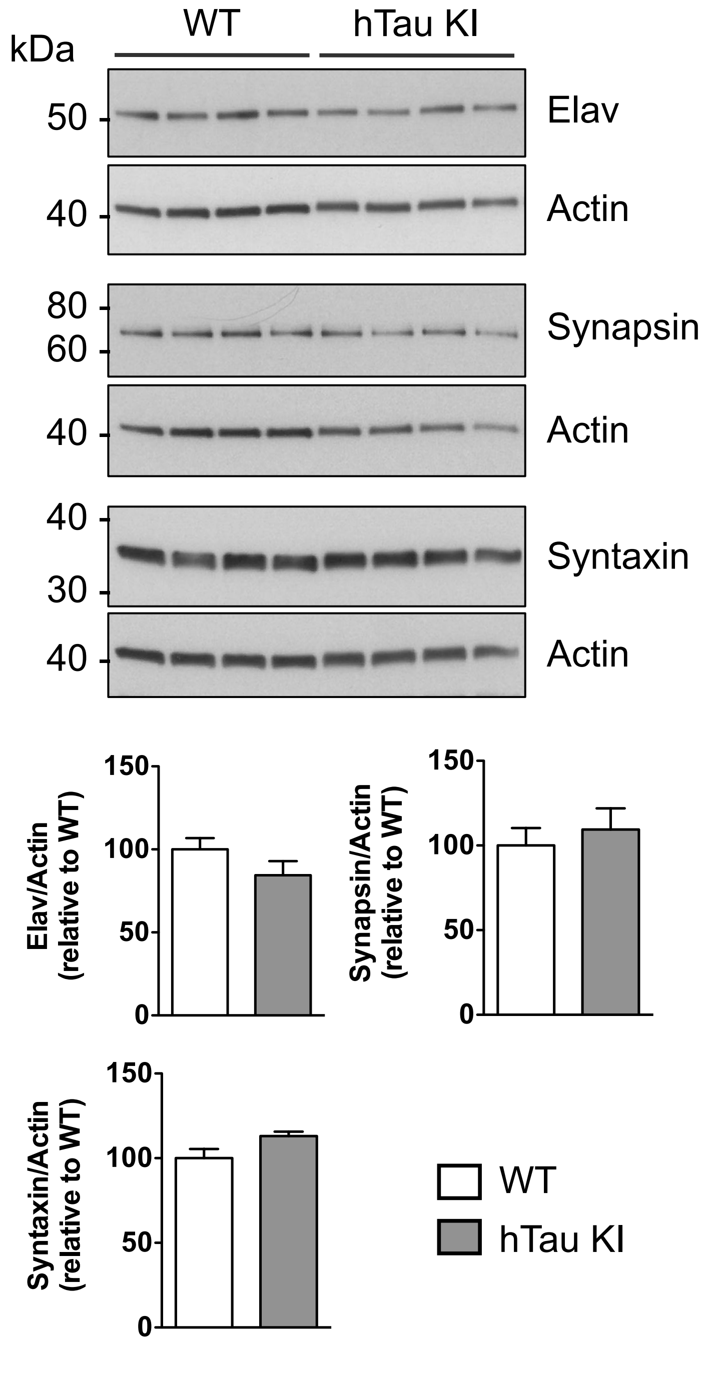

Supplementary Figure-6-Partridge. Schematic representation of hTau acetylation and phosphorylation sites investigated in this study.

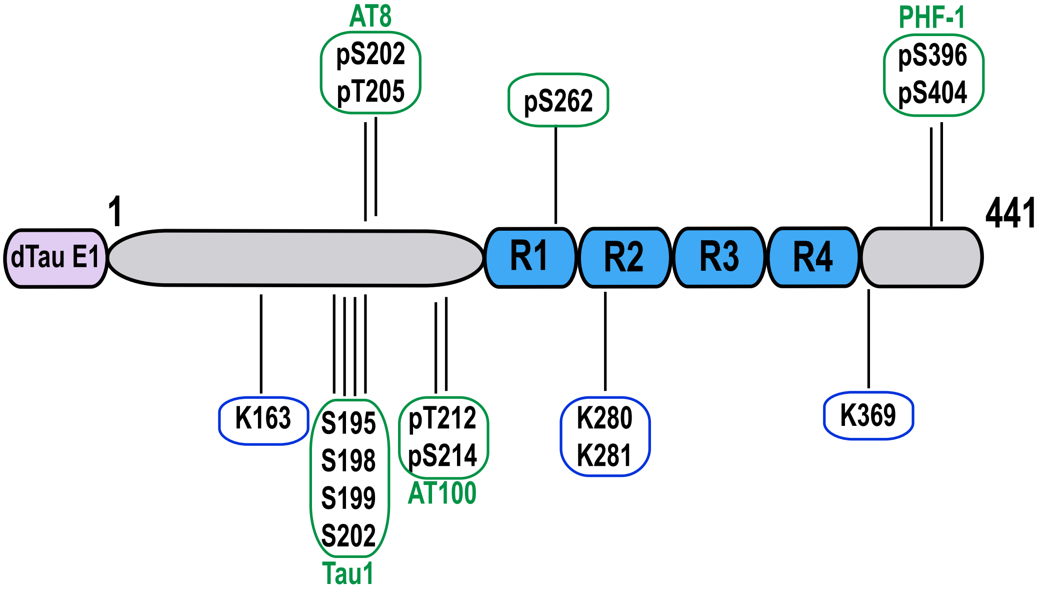

Supplementary Figure-7-Partridge. hTau mRNA levels were comparable in all hTau KI lines.

a

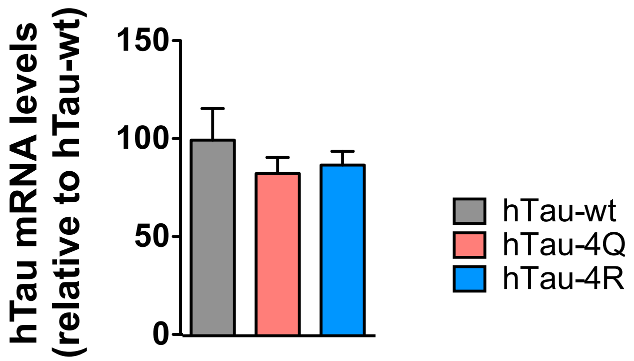

b

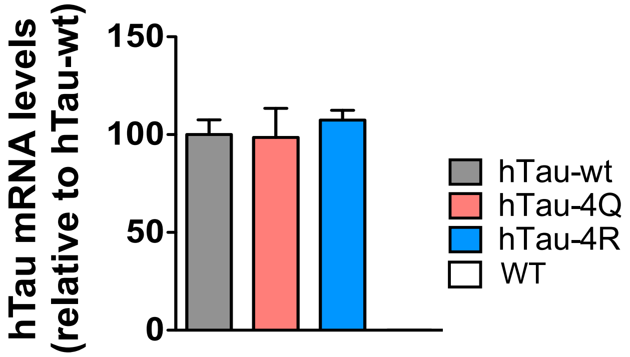

Supplementary Figure-8-Partridge. Imaging of hTau expression in hTau-wt, -4Q and -4R embryos and adult brains.

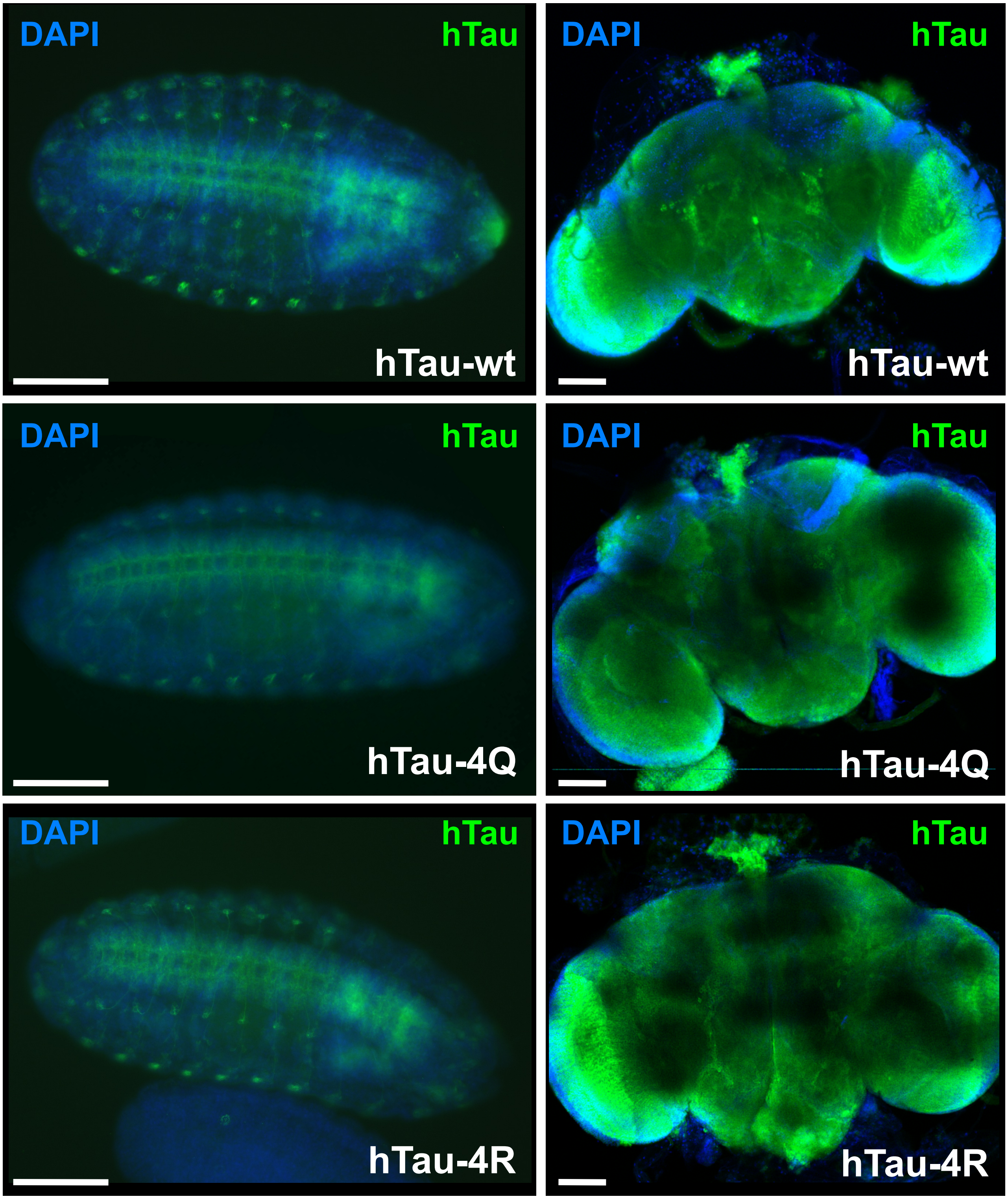

Supplementary Figure-9-Partridge. Mimicking multi-acetylation altered hTau phosphorylation pattern in 20-day-old flies.

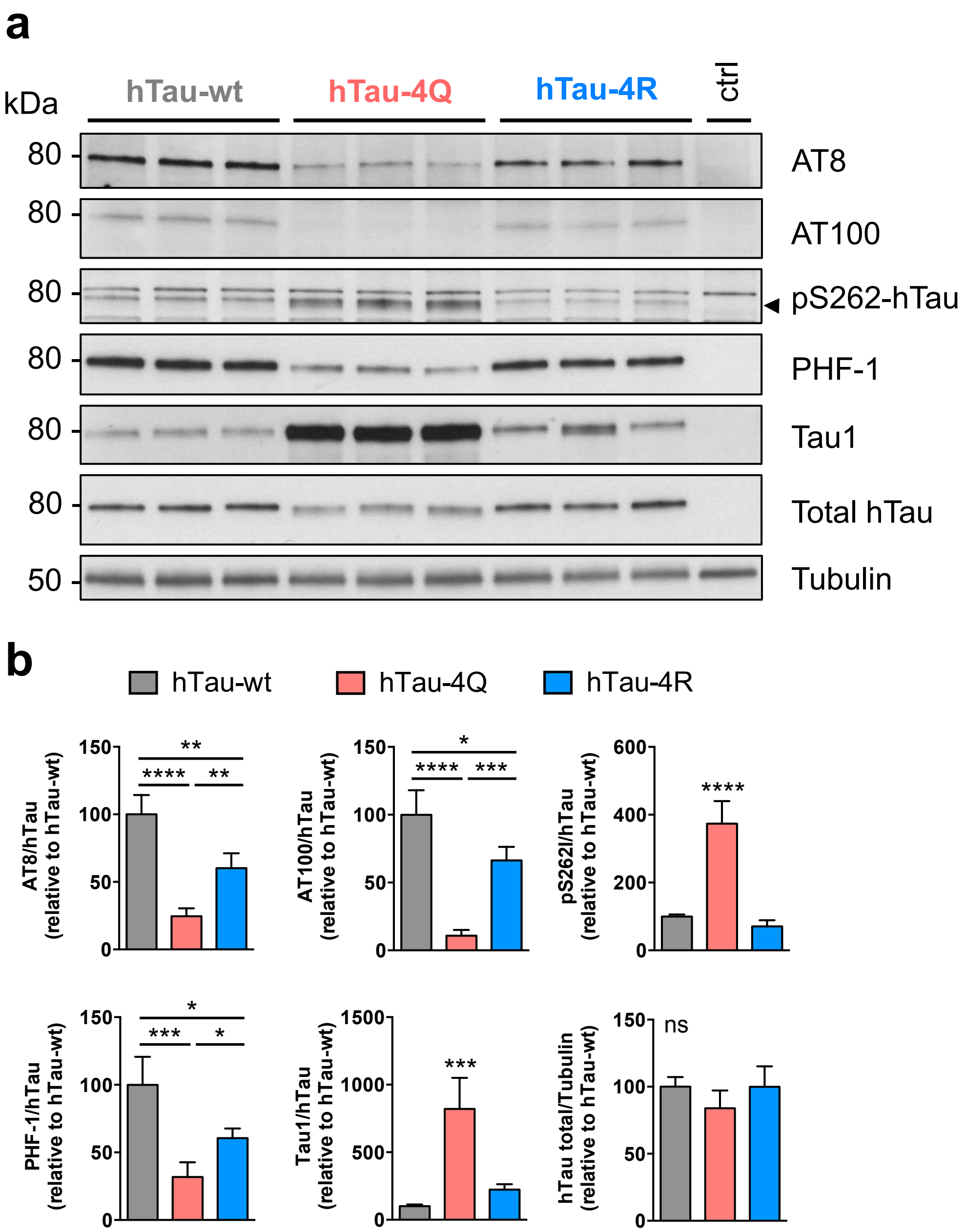

Supplementary Figure-10-Partridge. Evaluation of hTau phosphorylation pattern in hTau-wt, hTau-K280Q and hTau-K280R KI flies.

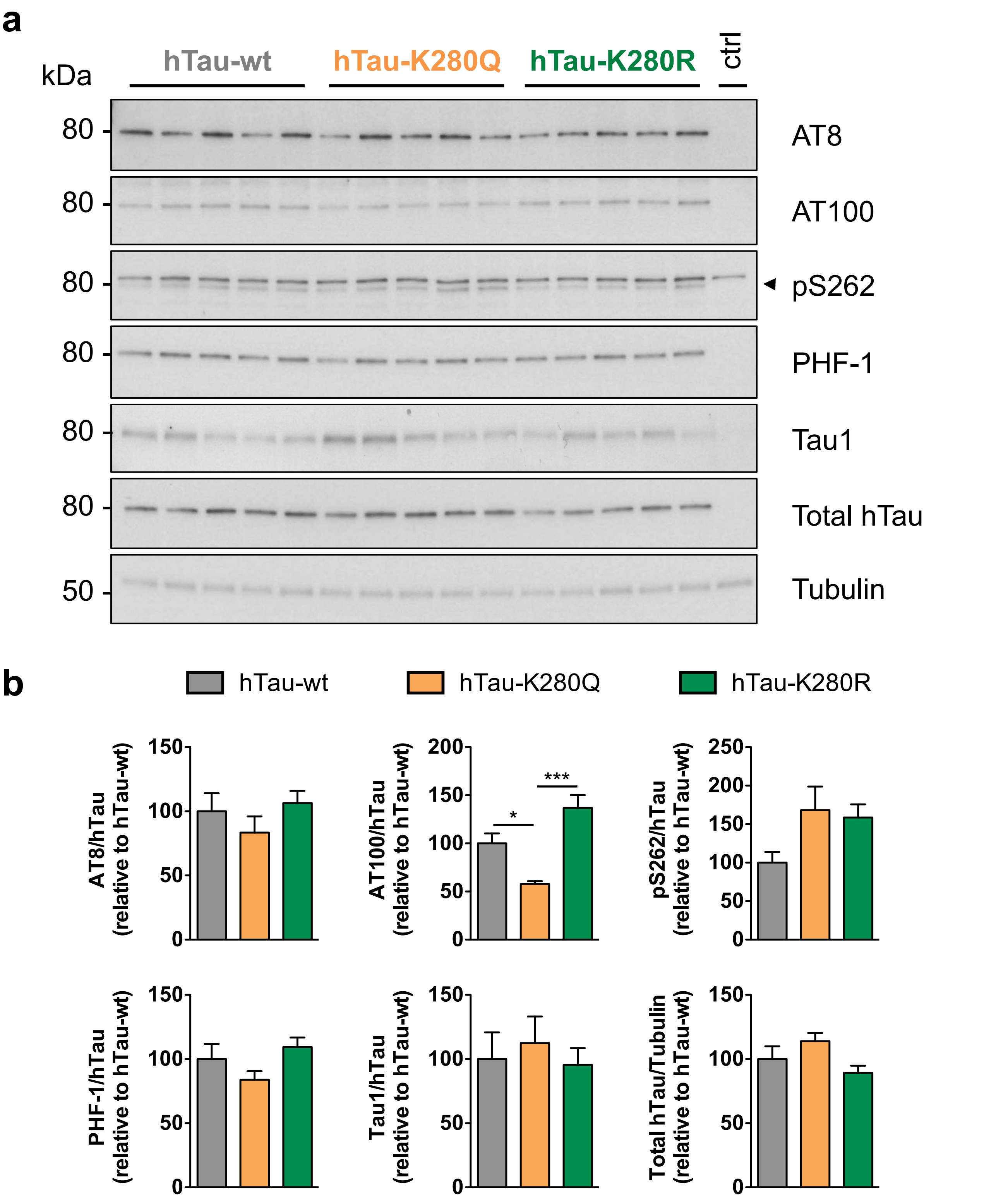

Supplementary Figure-11-Partridge. Mimicking multi-acetylation did not affect hTau solubility

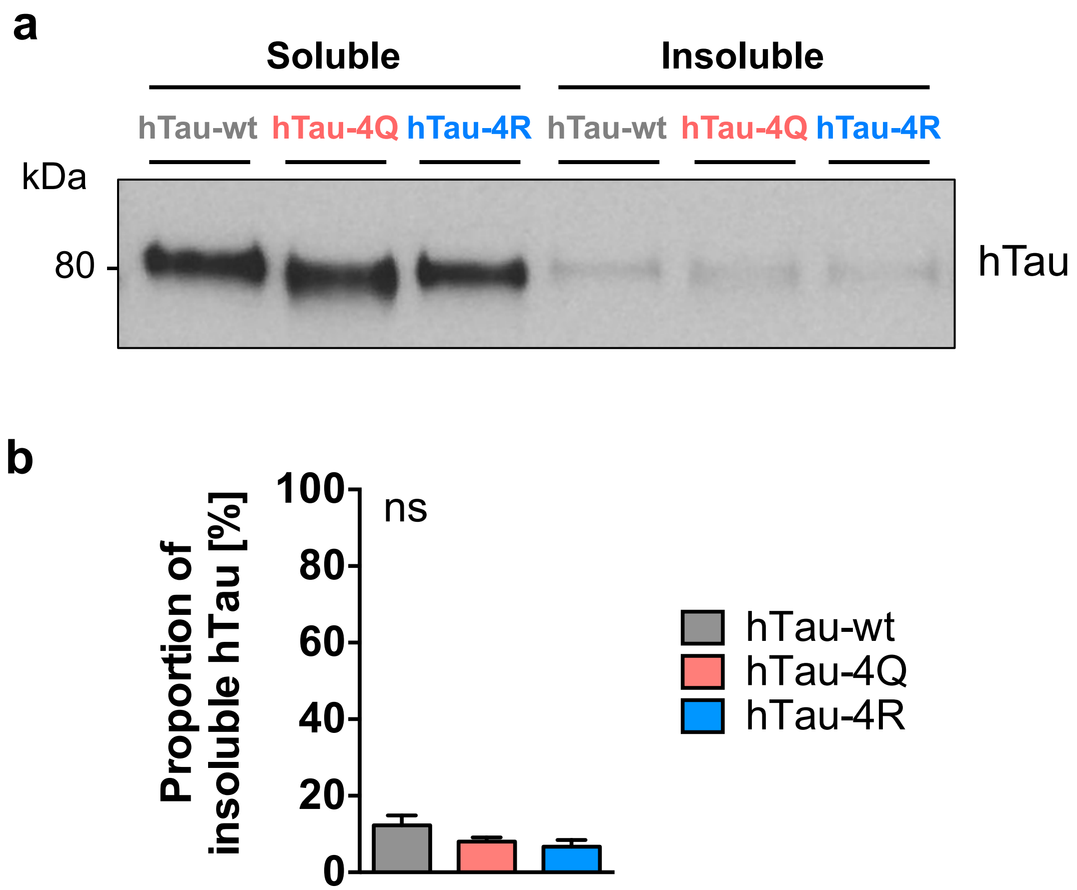

Supplementary Figure-12-Partridge. Evaluation of Tubulin acetylation in hTau-wt, -4Q and -4R KI flies.

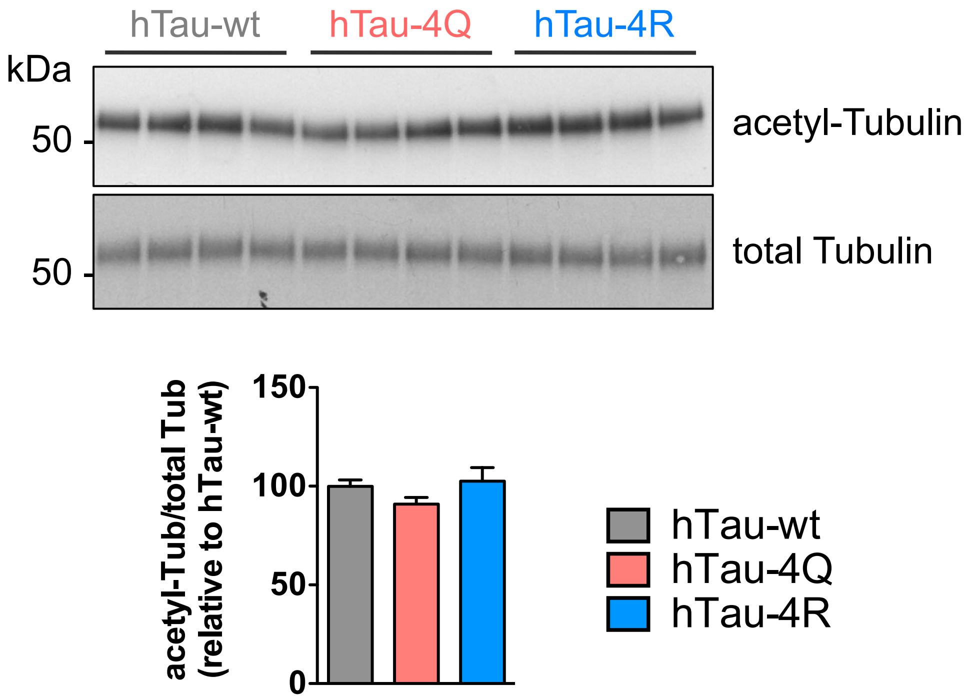

Supplementary Figure-13-Partridge. hTau-K280Q KI proteins displayed unaltered microtubule-binding properties

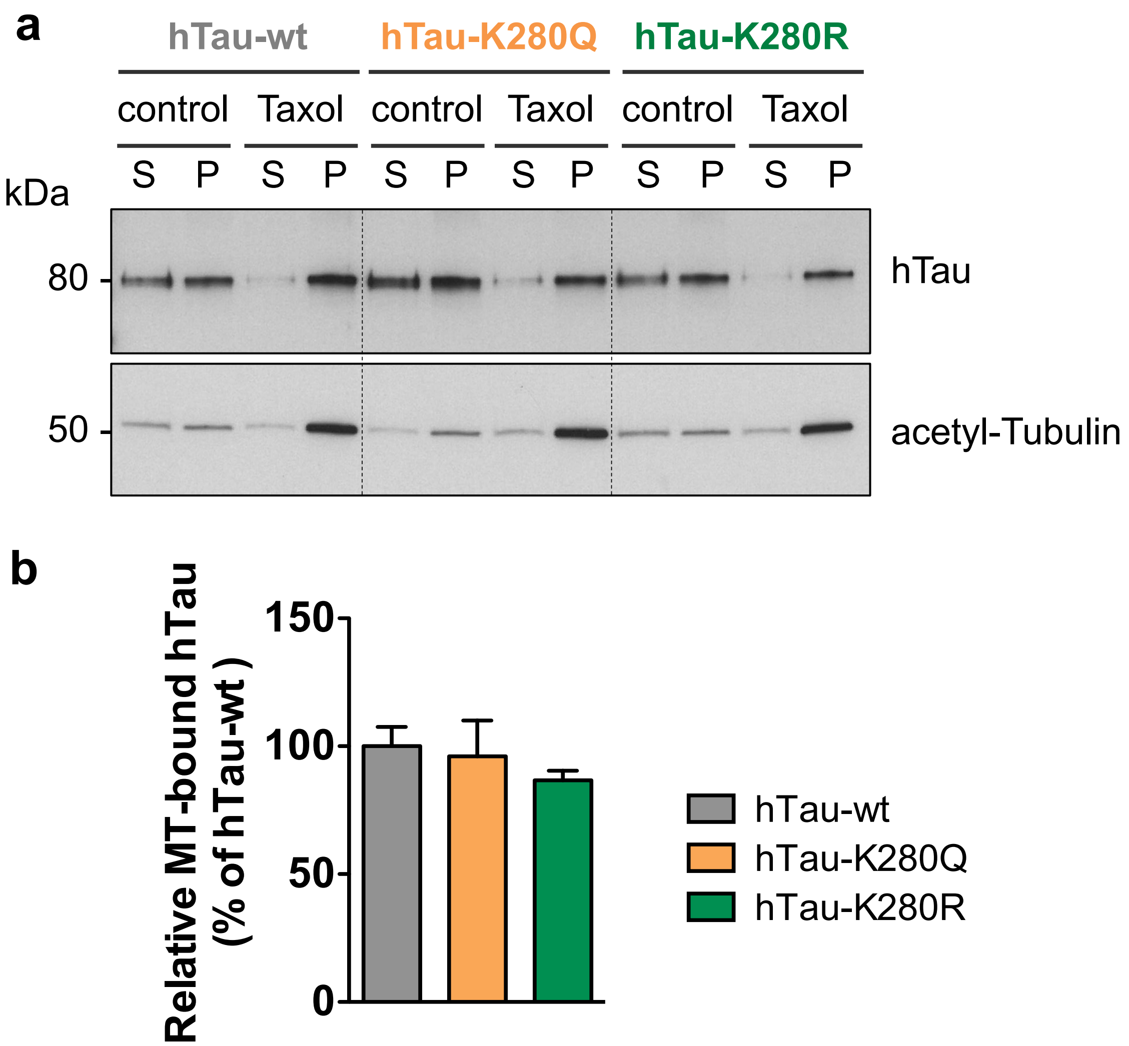

Supplementary Figure-14-Partridge. Evaluation of Aβ levels in flies co-expressing Aβ and homozygous hTau-wt, -4Q and -4R KI lines.

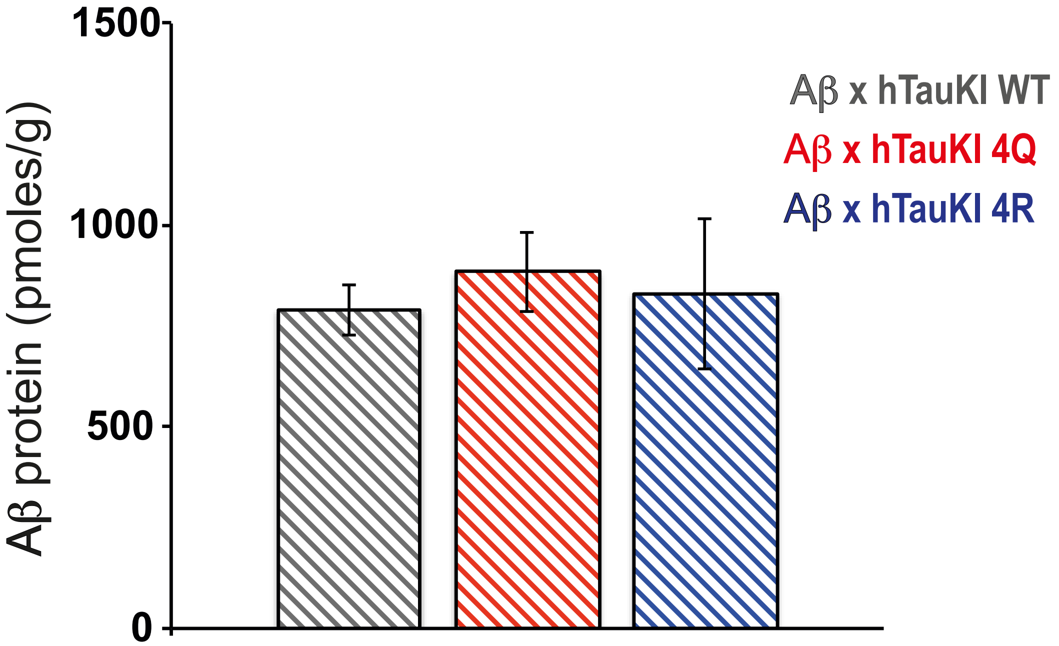

Supplementary figure 15 - Full blots from Figure 1

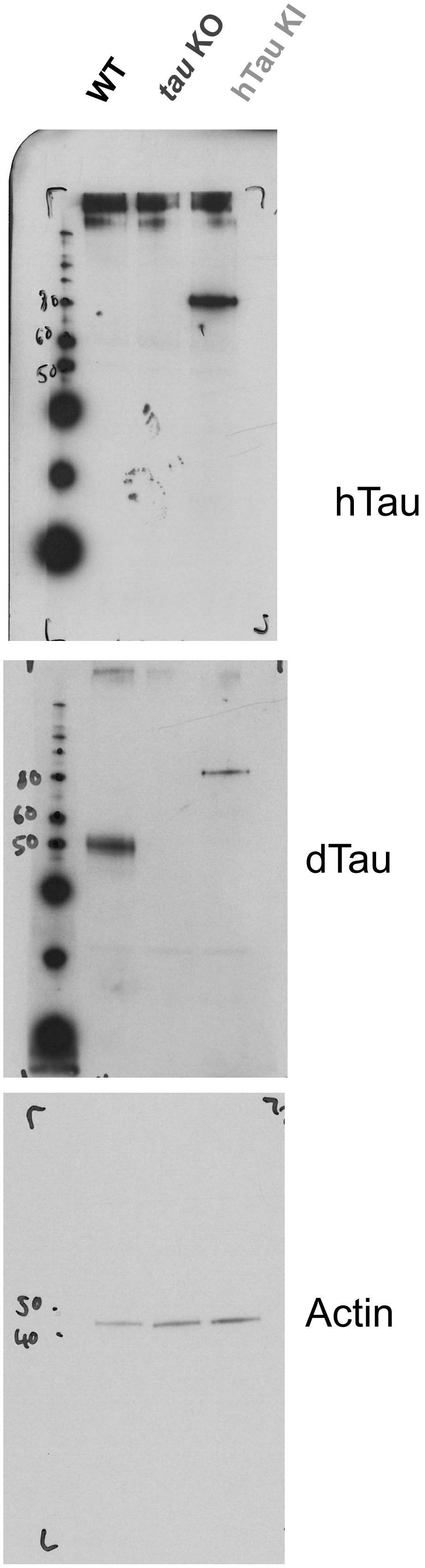

Supplementary figure 16 - Full blots from Figure 3

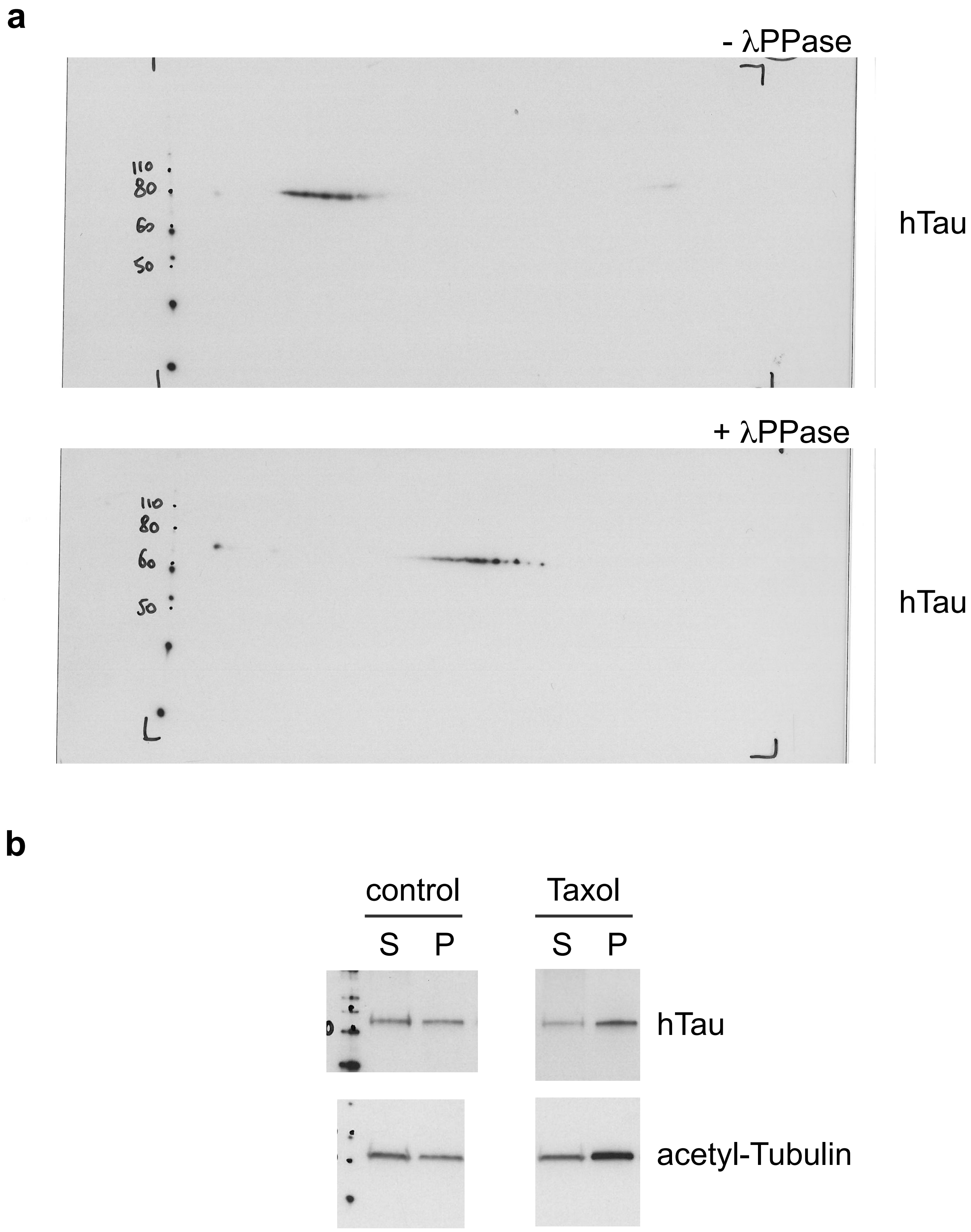

Supplementary figure 17 - Full blots from Figure 5

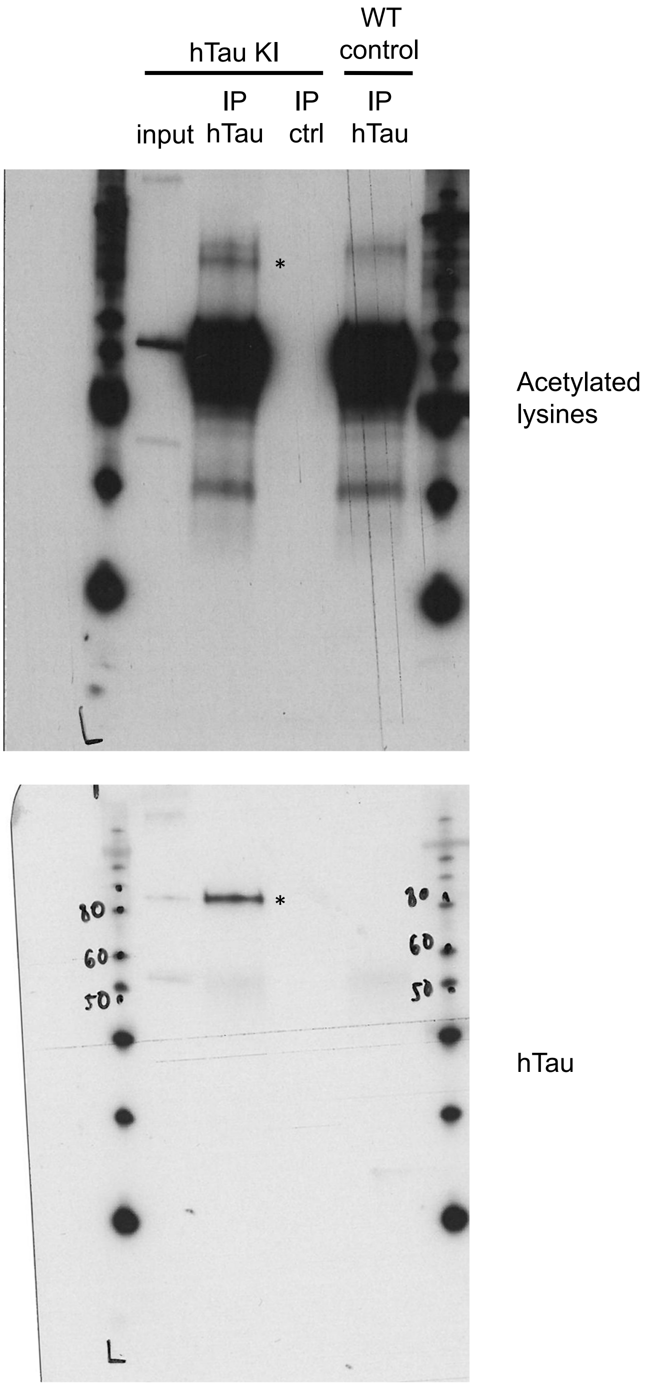

Supplementary figure 18 - blots from Figure 6

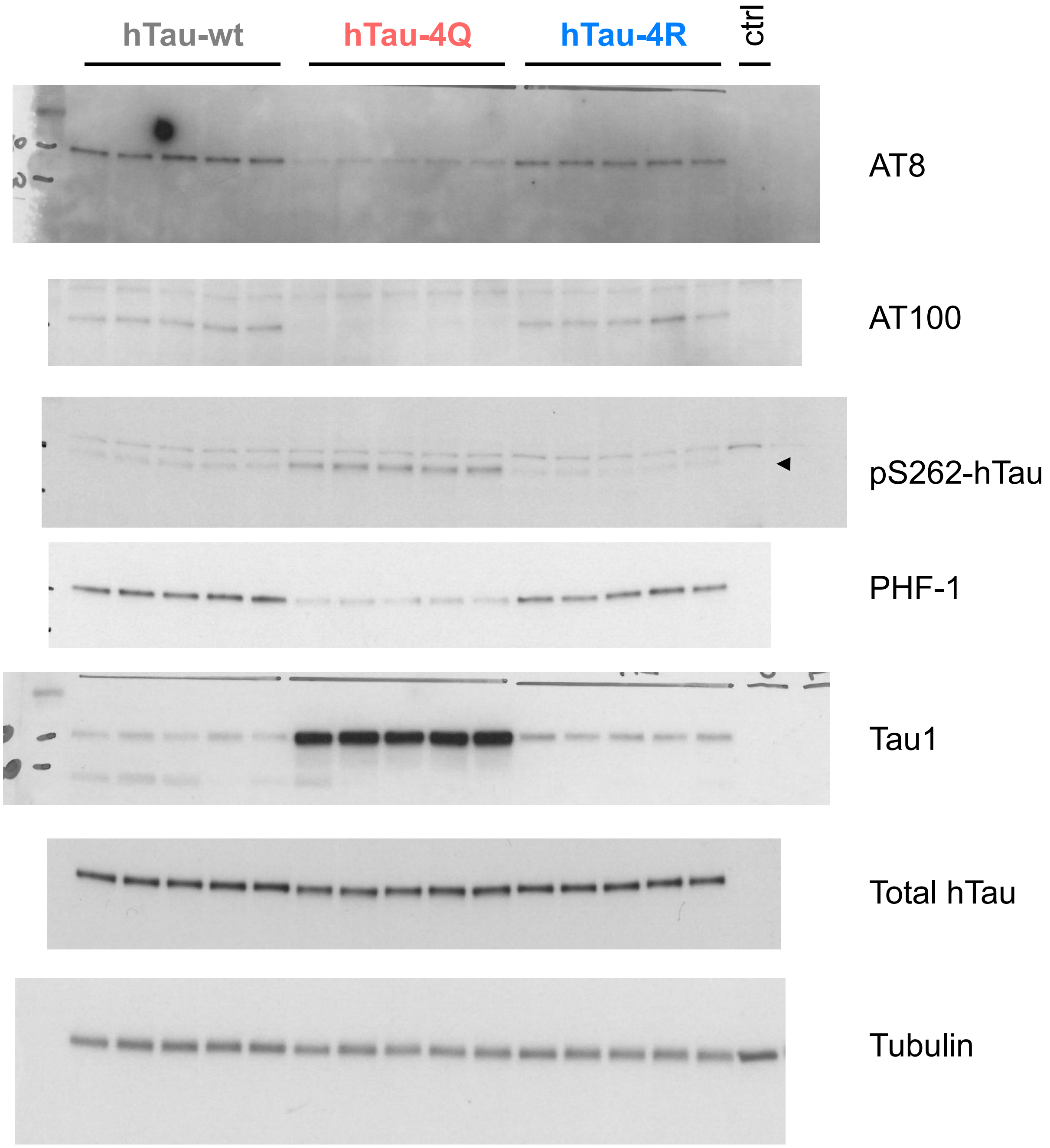

Supplementary figure 19 - Full blots from Figure 7

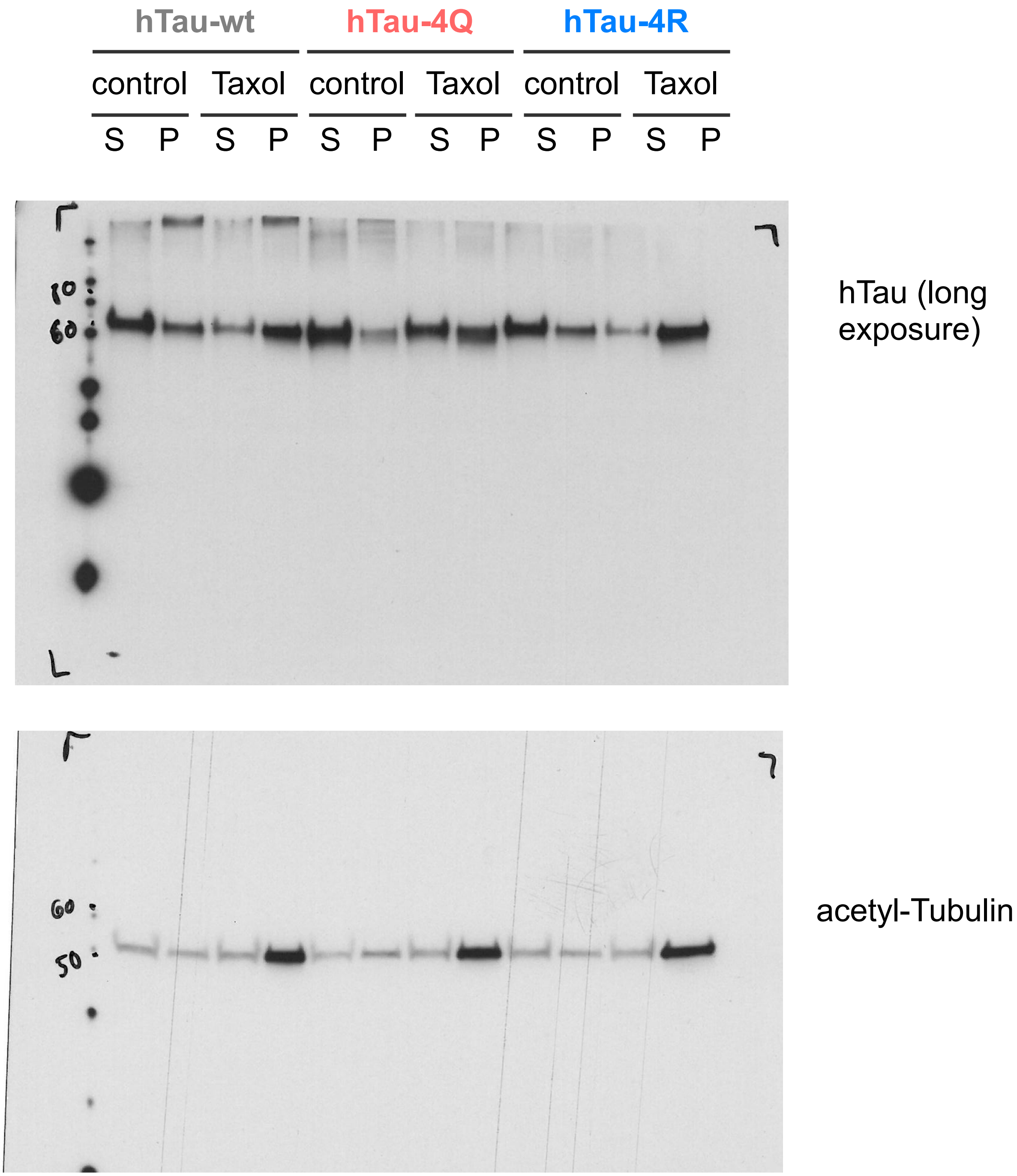

Supplementary figure 20 - blots from Figure 8

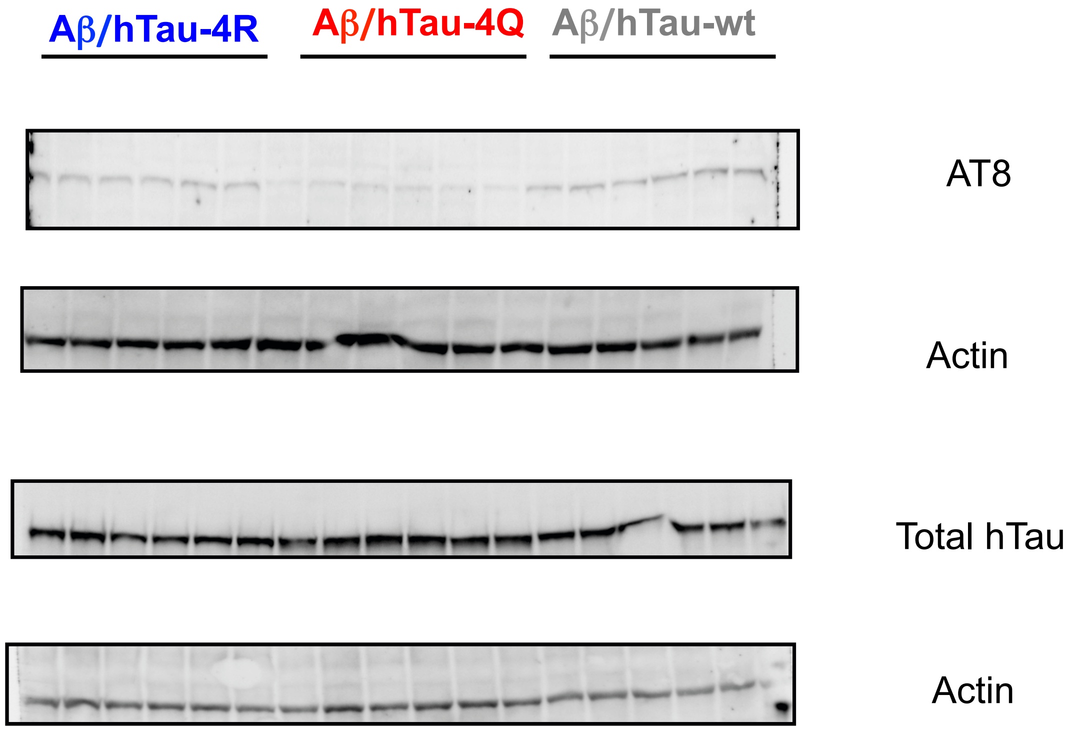

SUPPLEMENTARY FIGURE LEGENDS

Supplementary Figure 1. Genomic engineering of the fly *tau* locus. a. A genomic engineering strategy combining ends-out homologous recombination, the use of a Cre recombinase and φC31-mediated integration of replacement constructs was used to generate the hTau KI lines. Ampr: Ampicilline resistance gene. **b.** Nucleotide sequence of the *tau* replacement insert used to reconstitute the fly *tau* locus. The hTau 2N4R cDNA is indicated in grey while *Drosophila tau* sequences are shown in purple. The lysine residues investigated in this study are indicated in blue.

Supplementary Figure 2. Amino acid sequence of the hTau KI fusion protein. The hTau 2N4R protein isoform (441 amino acids, grey) is fused to fly *tau* exon 1 (82 amino acids, purple). Lysines 163, 280, 281 and 369 are indicated in blue. The hTau KI fusion protein has a theoretical MW of 54.67 kDa and runs at the apparent MW of 80 kDa.

Supplementary Figure 3. hTau KI proteins were expressed in brains of adult flies. a and b. Immunofluorescence for hTau and dTau was performed on brains of adult hTau KI (**a**) and WT (**b**) flies, respectively, indicating that hTau was localised to the nervous system in adult flies. DAPI was used to stain cell nuclei (blue). **c and d.** Immunofluorescence against hTau (**c**) and dTau (**d**) proteins was performed in adult *tau* KO control fly brains, showing no Tau immunoreactivity (green) in this line. DAPI was used to stain cell nuclei (blue). Scale bar: 100 µm.

Supplementary Figure 4. Control experiments for hTau immunofluorescence on fly embryos. Immunofluorescence on WT (**b**) and *tau* KO (**c**) control embryos showed no signal for hTau (green). The htau KI embryo is shown as a positive control (**a**). DAPI was used to stain cell nuclei (blue). Scale bar: 50 µm.

Supplementary Figure 5. Neuronal and synaptic protein levels were not altered in hTau KI flies. Expression of neuronal (Elav) and synaptic (Synapsin and Syntaxin) proteins was not impaired in 20-day-old hTau KI flies as compared to age-matched WT controls ($p>0.05$, Student's t-test). Results were normalised to Actin and expressed relative to levels measured in WT flies.

Supplementary Figure 6. Schematic representation of hTau acetylation and phosphorylation sites investigated in this study. hTau phosphorylation on S195/S198/S199/S202 (Tau1, binding to dephosphorylated hTau species) S202/T205 (AT8), T212/S214 (AT100), S262, and S396/S404 (PHF-1) was analysed following pseudo-acetylation or pseudo-deacetylation of hTau at K163, K280, K281 and K369 (hTau-4Q and hTau-4R, respectively).

Supplementary Figure 7. hTau mRNA levels were comparable in all hTau KI lines. hTau mRNA levels were measured by qRT-PCR in fly heads using a probe targeting either fly *tau* exon 1 (**a**), which is expressed in all investigated lines, or a common region on hTau sequence (**b**). WT flies are shown as a negative control for hTau expression. hTau-wt, -4Q and -4R KI flies expressed comparable levels of hTau transcripts ($p>0.05$, one-way ANOVA).

Supplementary Figure 8. Imaging of hTau expression in hTau-wt, -4Q and -4R embryos and adult brains. hTau immunofluorescence (green) was performed on embryos (left panels) and adult brains (right panels) of hTau-wt, hTau-4Q and hTau-4R KI flies. DAPI was used to stain cell nuclei (blue). Scale bars: 100 µm.

Supplementary Figure 9. Mimicking multi-acetylation altered hTau phosphorylation pattern in 20-day-old flies. Western blot analysis (**a**) and quantification (**b**) of hTau phosphorylation (AT8, AT100, anti-pS262-hTau, PHF-1 and Tau1 antibodies) and total hTau levels (polyclonal K9JA antibody) retrieved from head extracts of 20-day-old hTau-wt, hTau-4Q and hTau-4R KI flies. Results were normalized to Tubulin and to total hTau levels and expressed relative to the hTau-wt KI line (*$p<0.05$, **$p<0.01$, ***$p<0.001$ and ****$p<0.0001$, one-way ANOVA followed by Tukey's post hoc test, n=3/genotype). Although total hTau levels retrieved from hTau-4Q fly heads appeared lower, imageJ quantification revealed similar hTau protein levels among the three mutants ($p>0.05$, one-way ANOVA), the corresponding band being simply more diffuse in hTau-4Q samples.

Supplementary Figure 10. Evaluation of hTau phosphorylation pattern in 2-day-old hTau-wt, hTau-K280Q and hTau-K280R KI flies. Western blot analysis (**a**) and quantification (**b**) of hTau phosphorylation (AT8, AT100, anti-pS262-hTau, PHF-1 and Tau1 antibodies) and total hTau levels (polyclonal K9JA antibody) retrieved from head extracts of 2-day-old hTau-wt, hTau-K280Q and hTau-K280R KI flies. Results were normalized to Tubulin and to total hTau levels and expressed relative to the hTau-wt KI line (*$p<0.05$ and ***$p<0.001$, one-way ANOVA followed by Tukey's post hoc test, n=5/genotype).

Supplementary Figure 11. Mimicking multi-acetylation did not affect hTau solubility. a. Protein extracts from hTau KI fly heads were fractionated into RAB-soluble and RAB-insoluble/RIPA-1%SDS-soluble fractions and subsequently probed with an anti-hTau antibody (K9JA). **b.** Quantification of the proportion of insoluble hTau species observed in fly head extracts from hTau-wt, hTau-4Q and hTau-4R KI lines ($p>0.05$, one-way ANOVA followed by Tukey's post hoc test, n=4/genotype).

Supplementary Figure 12. Evaluation of Tubulin acetylation in hTau-wt, -4Q and -4R KI flies. Western blot analysis indicated that levels of acetylated Tubulin retrieved from hTau-wt, hTau-4Q and hTau-4R KI fly heads were comparable ($p>0.05$, one-way ANOVA). Results were normalised to total Tubulin and expressed relative to levels measured in hTau-wt KI flies.

Supplementary Figure 13. hTau-K280Q KI proteins displayed unaltered microtubule-binding properties. a. In vivo microtubule-binding assay was performed on protein extracts from 2-day-old hTau-wt, hTau-K280Q and hTau-K280R KI fly heads. Taxol was used to induce microtubule polymerisation, and DMSO, the vehicle, was used as a control. S: supernatant, P: pellet. **b.** Quantification of the proportion of microtubule-bound hTau proteins in hTau-wt, hTau-K280Q and hTau-K280R KI lines, in Taxol conditions. Results are expressed relative to levels measured in hTau-wt KI flies ($p>0.05$, one-way ANOVA followed by Tukey's post hoc test, n=3/genotype).

Supplementary Figure 14. Aβ levels did not change across the different hTauKI lines. Flies co-expressing arctic Aβ42 in combination with any of the homozygous hTau-wt, hTau-4Q and hTau-4R KI lines had similar levels of Aβ42 protein by ELISA analyses, ($p>0.05$, one-way ANOVA followed by Tukey's post hoc test, n=4/genotype).

Supplementary Figure 15. Full blots from Figure 1.

Supplementary Figure 16. Full blots from Figure 3.

Supplementary Figure 17. Full blots from Figure 5.

Supplementary Figure 18. Full blots from Figure 6.

Supplementary Figure 19. Full blots from Figure 7.

Supplementary Figure 20. Full blots from Figure 8.
